# Combinatorial profiling of multiple histone modifications and transcriptome in single cells using scMTR-seq

**DOI:** 10.1126/sciadv.adu3308

**Published:** 2025-08-06

**Authors:** Yang Wang, Jingyu Li, Andrew A. Malcolm, William Mansfield, Stephen J. Clark, Ricard Argelaguet, Laura Biggins, Richard J. Acton, Simon Andrews, Wolf Reik, Gavin Kelsey, Peter J. Rugg-Gunn

**Affiliations:** ^1^ Epigenetics Programme, Babraham Institute, Cambridge CB22 3AT, UK.; ^2^ Bioinformatics Group, Babraham Institute, Cambridge CB22 3AT, UK.; ^3^ Loke Centre for Trophoblast Research, University of Cambridge, Cambridge CB2 3EG, UK.; ^4^ Wellcome Sanger Institute, Wellcome Genome Campus, Cambridge CB10 1RQ, UK.; ^5^ Institute of Metabolic Science-Metabolic Research Laboratories, University of Cambridge, Cambridge CB2 0QQ, UK.; ^6^ Cambridge Stem Cell Institute, Jeffrey Cheah Biomedical Centre, University of Cambridge, Cambridge CB2 0AW, UK.

## Abstract

Profiling combinations of histone modifications identifies gene regulatory elements in different states and discovers features controlling transcriptional and epigenetic programs. However, efforts to map chromatin states in complex, heterogeneous samples are hindered by the lack of methods that can profile multiple histone modifications together with transcriptomes in individual cells. Here, we describe single-cell multitargets and mRNA sequencing (scMTR-seq), a high-throughput method that enables simultaneous profiling of six histone modifications and transcriptome in single cells. We apply scMTR-seq to uncover dynamic and coordinated changes in chromatin states and transcriptomes during human endoderm differentiation. We also use scMTR-seq to produce lineage-resolved chromatin maps and gene regulatory networks in mouse blastocysts, revealing epigenetic asymmetries at gene regulatory regions between the three embryo lineages and identifying Trps1 as a potential repressor in epiblast cells of trophectoderm-associated enhancer networks and their target genes. Together, scMTR-seq enables investigation of combinatorial chromatin landscapes in a broad range of heterogeneous samples, providing insights into epigenetic regulatory systems.

## Teaser

scMTR-seq enables joint profiling of multiple histone modifications and transcriptome to study epigenome dynamics and regulation.

## INTRODUCTION

Advances in chromatin profiling technologies have provided opportunities to investigate epigenetic mechanisms in complex and heterogeneous cellular systems (*1*, *2*). Combinations of multiple histone modifications are required to categorize gene regulatory elements, including different forms of repressed, poised, and active configurations of enhancers and promoters (*3*–*5*). Such differences in regulatory activity are often indistinguishable when using single-modality profiles alone. To more accurately decipher a broad range of epigenetic inputs, it is necessary to simultaneously profile multiple chromatin modalities within individual cells.

Methods that profile histone modifications in single cells use different strategies, such as antibody cross-linking with indexed proteinA-Tn5, or sequential tagmentation with different antibody-indexed proteinA-Tn5, or nanobody-mediated indexed Tn5 that target specific species or antibody isotypes (*6*–*19*). These approaches allow antibody-specific index tagmentation at targeted genomic loci, whereby each antibody recognizes a distinct histone modification (*6*–*19*). These approaches provide a first step toward mapping more than one histone modification in single cells. However, methods that rely on sequential tagmentation steps for each histone modification, although relatively straightforward to implement, require substantial hands-on time typically over several days, and each round of tagmentation risks sample deterioration that compromises library quality and yield. Sample loss during library preparation further reduces cell recovery and necessities a large number of cells in the starting material, commonly hundreds of thousands of cells, which makes these methods incompatible with many samples. Moreover, there is a lack of high-throughput methods that can simultaneously profile multiple histone modifications together with transcriptome in the same individual cell with high specificity and sensitivity. Overcoming this limitation would enable accurate definition of cell-type–resolved chromatin state maps that rely on combinations of chromatin modifications.

Here, we present scMTR-seq (single-cell multitargets and mRNA sequencing), which is a multiomics sequencing technology that can simultaneously profile multiple histone modifications and transcriptome in the same single cells with high sensitivity and high cell recovery and is highly scalable in terms of starting material requirement.

## RESULTS

### Combined profiling of multiple histone modifications and transcriptome modalities

**Fig. 1.**
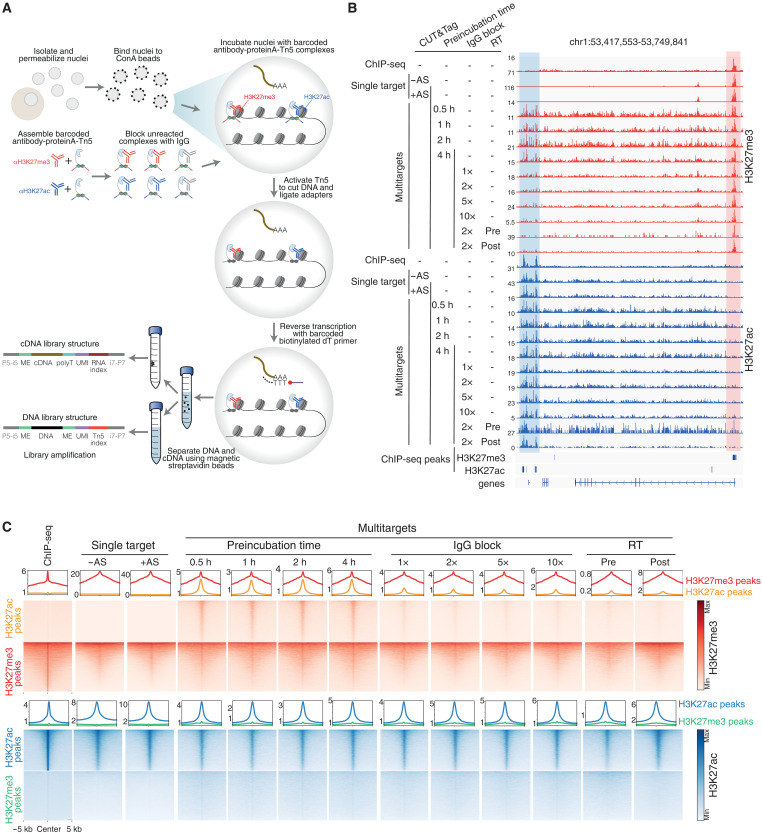
Development of method to jointly profile transcriptome and multiple histone modifications. (**A**) Overview of main steps in method. ME, mosaic end; UMI, unique molecular identifiers. (**B**) Genome browser tracks show H3K27me3 (red) and H3K27ac (blue) signals in human pluripotent stem cells (hPSCs) under various conditions tested. Tracks compare ENCODE chromatin immunoprecipitation sequencing (ChIP-seq), single-modality CUT&Tag, and our multimodality method (H3K27me3 and H3K27ac, with and without joint transcriptome profiling). AS, adapter switching. Preincubation time refers to the duration when the antibody-proteinA-Tn5-adapter complexes were combined. IgG block refers to the amount of IgG antibody used to block any unreacted protein A-Tn5. RT, reverse transcription step, where “pre” means transcriptome profiling before the Tn5 tagmentation step and “post” means after Tn5 tagmentation. (**C**) Line plots and heatmaps show the same data as in (B) for all H3K27me3 peaks and all H3K27ac peaks defined by ENCODE ChIP-seq datasets. Top: Line plots show average H3K27me3 signal in ENCODE H3K27me3 peaks (red lines) and in ENCODE H3K27ac peaks (orange lines), and heatmaps in red coloring show H3K27me3 signal. Bottom: Line plots show average H3K27ac signal in ENCODE H3K27me3 peaks (green lines) and in ENCODE H3K27ac peaks (blue lines), and heatmaps in blue coloring show H3K27ac signal.

To profile multiple histone modifications and transcriptome in the same cells, we developed a method with the following steps: (i) preassemble antibodies specific for each target histone modification with indexed proteinA-Tn5-adapters; (ii) perform in situ Tn5-mediated tagmentation with indexed complexes; (iii) capture nuclear mRNA with a barcoded poly-T primer followed by in situ reverse transcription (RT) (Fig. 1A and table S1). We performed a series of optimization steps to increase the robustness and sensitivity of the method, as described below.

First, we implemented an adapter switching strategy (*20*) that uses the mosaic end B (MEB) adapter for antibody-specific tagmentation and then adds the mosaic end A (MEA) adapter to all MEB-tagged fragments. Including this step did not affect the profiled localization of the histone modifications (Fig. 1, B and C; and fig. S1, A and B), and it improved the signal-to-background ratio, as shown by the higher fraction of reads in peaks (FRiP) (fig. S1C). Furthermore, there were more unique reads in libraries prepared with adapter-switching compared to without, indicating that these libraries achieved higher library complexity (fig. S1D).

Second, to assess the performance of our method in mapping multiple histone modifications, we compared different conditions for simultaneously profiling H3K27me3 and H3K27ac in human pluripotent stem cells (hPSCs). H3K27me3 and H3K27ac peaks were successfully captured [H3K27me3, coefficient of determination (*R*^2^) = ~0.98; H3K27ac, *R*^2^ = ~0.90, when compared to individual profiles] (Fig. 1, B and C, and figs. S1A and S2A). However, consistent with previous reports (*11*), directly combining multiple preassembled antibody-proteinA-Tn5-adapter complexes to simultaneously tagment cells resulted in off-target signals, as H3K27ac-assigned reads aberrantly overlapped with H3K27me3 signal, a problem that was not alleviated in conditions with extended preincubation times (Fig. 1, B and C). Hypothesizing that off-target signals might be caused by excess, free proteinA after assembly with histone modification antibodies, we found that adding immunoglobulin G (IgG) blocking antibodies to the postassembled proteinA-antibody mixture strongly reduced off-target signals (Fig. 1, B and C, and figs. S1A and S2B) and increased the FRiP (fig. S1C). Although adding more IgG slightly helped to further improve the detection of H3K27me3 and H3K27ac enriched signals at on-target peaks (figs. S1C and S2C), it did not further reduce off-target signals (Fig. 1, B and C, and figs. S1A and S2B).

Third, to capture transcriptome information together with multiple histone modifications, we assessed whether transcriptome profiling and chromatin profiling would affect each other. Performing RT of RNA before DNA tagmentation or after DNA tagmentation did not affect transcriptome profiles or sensitivity (fig. S3, A and B). However, RT before DNA tagmentation was detrimental to chromatin profiling, resulting in higher background signal (Fig. 1B and figs. S1A and S2, B and C). We therefore performed RT after DNA tagmentation in subsequent experiments. Together, we have established an efficient method for jointly profiling transcriptome and histone modifications, with low background signal and good reproducibility.

### scMTR-seq profiles six targets and transcriptome in single cells

**Fig. 2.**
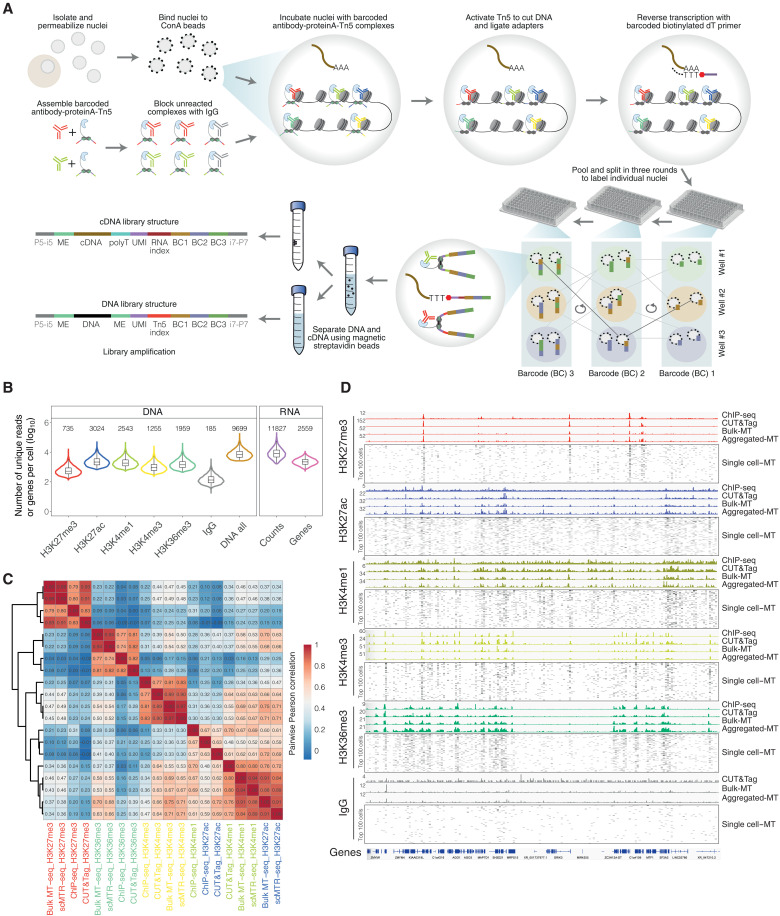
scMTR-seq jointly profiles multiple histone modifications and transcriptomes in single cells. (**A**) Overview of scMTR-seq steps. ME, mosaic end; UMI, unique molecular identifiers; BC, barcode. (**B**) Violin plot of unique reads per cell for the six targets profiled and of unique RNA counts plus the number of genes per cell for the transcriptome. Numbers above each dataset shows mean values. Boxplots show median with interquartile range and minimum/maximum whiskers. (**C**) Heatmaps show pairwise Pearson correlation between datasets from ENCODE ChIP-seq, single-target CUT&Tag, bulk sample multitarget CUT&Tag (six targets; “Bulk-MT”), and computationally aggregated scMTR-seq (six targets) assays using genome-wide signals of 5-kb bins. The numbers show the Pearson correlation coefficients. (**D**) Genome browser tracks show histone modification signals for ENCODE ChIP-seq, single-target CUT&Tag, Bulk-MT, and computationally aggregated scMTR-seq (six targets; “Aggregated MT”). scMTR-seq profiles are also shown for individual cells (100 cells per histone modification and IgG; “Single cell–MT”).

We next sought to combine multiomic profiling with a scalable and high-throughput approach applicable to single cells. To achieve this, following in situ DNA tagmentation and RT on intact nuclei, we applied three rounds of split-pool combinatorial barcoding (Fig. 2A) (*21*–*23*). With 48 unique barcodes for each barcoding round, more than 110,000 unique barcode combinations can be generated after three rounds of barcoding, which is sufficient to uniquely label 1100 to 11,000 single cells with an estimated collision rate of 1 to 10%, respectively. Although this barcoding capacity was sufficient for our experiments, capacity could be scaled up to millions of cells by using multiple sublibraries or by increasing the number of barcodes per round.

To test the robustness of our method, we applied scMTR-seq to simultaneously profile six targets (H3K4me1, H3K4me3, H3K27ac, H3K27me3, H3K36me3, and IgG) and transcriptome in hPSCs. Starting with 18,000 cells, we obtained combined histone modification and transcriptome data on 7479 cells after applying quality control filters for DNA (unique reads per cell > 2500) and RNA (unique reads per cell > 2000), thereby achieving a high recovery rate of >40% cells (fig. S4A and table S2). With a sequencing depth of 75,000 total raw reads per cell for DNA and 22,000 raw reads per cell for RNA, we obtained on average 9699 unique reads per cell for DNA (ranging from a mean of 735 to 3024 reads for different histone modifications) and 11,827 unique reads with 2559 genes per cell for RNA (Fig. 2B and fig. S4B). Duplication rates were similar for all targets (fig. S4C). The FRiP varied for each histone modification, likely due to different signal to background ratios depending on the modification that was profiled (fig. S4D). As expected, the IgG sample had the lowest library complexity (Fig. 2B and fig. S4, B and C). Benchmarking scMTR-seq to other single-cell methods for profiling histone modifications revealed that scMTR-seq had a higher or comparable number of nonduplicated sequence reads per cell compared to Multi-CUT&Tag (*17*), nanobody-tethered transposition sequencing (NTT-seq) (*18*), MulTi-Tag (*11*), uCoTarget (*16*), and MAbID (*19*) and a higher number of genes per cell for RNA compared to uCoTargetX (*16*) (fig. S4E).

To characterize the scMTR-seq data, we computationally aggregated the single-cell profiles for each histone modification individually. Comparing aggregated scMTR-seq data with CUT&Tag data revealed strong correlation for all histone modifications profiled [H3K27me3, Pearson’s correlation coefficient (*r*) = 0.91; H3K36me3, *r* = 0.82; H3K4me3, *r* = 0.90; H3K4me1, *r* = 0.80; and H3K27ac, *r* = 0.69] and also with ENCODE chromatin immunoprecipitation sequencing (ChIP-seq) data (*r* = 0.59 to 0.83; Fig. 2C). Peaks identified using scMTR-seq overlapped with peaks defined by CUT&Tag and ChIP-seq (Fig. 2D and fig. S4, F to H). Visualizing genome tracks confirmed that each histone modification profiled by scMTR-seq was enriched at distinct domains including promoters (H3K4me3 and H3K27me3) and gene bodies (H3K36me3; Fig. 2D and fig. S4I). Increasing the number of single cells included in the aggregated scMTR-seq dataset helped to more accurately recapitulate bulk assay profiles, although this improvement started to level off above ~500 cells (fig. S4, J and K). Thus, combining the profiles of relatively few single cells is sufficient to reproduce large-scale bulk datasets.

Comparing signal specificity of scMTR-seq with that of other methods confirmed that all methods could detect good on-target signals, but CoTarget (*16*), NTT-seq (*18*), and MulTi-Tag (*11*) had higher levels of off-target signals compared to scMTR-seq, particularly at regions of active and accessible chromatin (fig. S5, A and B). We could further reduce off-target signals in scMTR-seq by taking advantage of the integrated IgG signals for data normalization (fig. S5A). To identify the source of low-level off-target signals in scMTR-seq, we found that a subset of peaks called in scMTR-seq, but not in CUT&Tag, coincided with regions of high H3K27ac and H3K4me1, suggesting that off-target signals in scMTR-seq arise mainly at regions of strong active chromatin (fig. S5C). Together, these results show that scMTR-seq compares favorably with other methods and achieves high specificity even with increased multiplexing capacity and the integrated capture of transcriptome profiles.

We next calculated Cramér’s V (*11*) to assess the relationship between each pair of histone modifications and with transcription in single cells. As expected, gene expression was positively associated with active histone marks, and H3K27me3 had low association with active histone marks and with gene expression (fig. S6A). We then used ChromHMM (*24*) to assess whether our scMTR-seq chromatin profiles faithfully identify chromatin states with different histone modification combinations. Integrating computationally aggregated data from the five profiled histone modifications led to the identification of seven chromatin states: active promoter, bivalent promoter, active enhancer, primed enhancer, transcription, Polycomb-associated and unmarked/unknown states (fig. S6B). These chromatin profiles successfully recapitulated >86% of the chromatin states that were assigned using conventional, single target CUT&Tag (fig. S6C). These results demonstrate that scMTR-seq faithfully profiles multiple chromatin modalities and transcriptome with high sensitivity and accuracy.

### scMTR-seq uncovers chromatin state dynamics during definitive endoderm differentiation

**Fig. 3.**
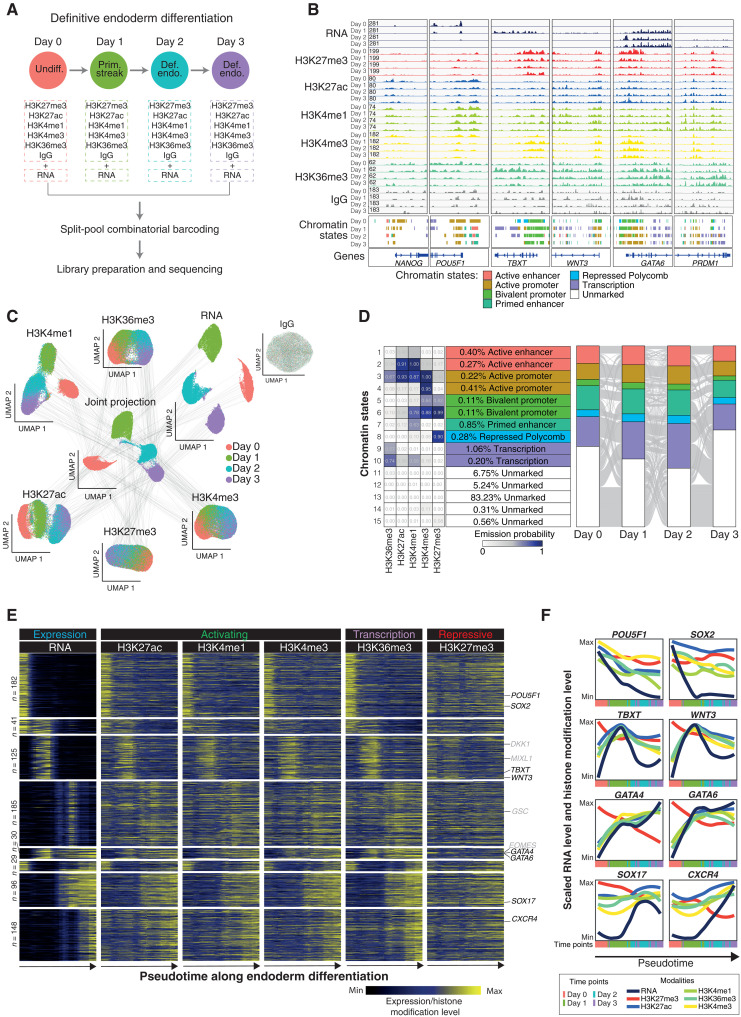
Chromatin state dynamics during human endoderm differentiation. (**A**) Experimental outline. (**B**) Genome browser tracks show changes in histone modifications, chromatin states and transcriptome at six candidate gene loci over hPSC endoderm differentiation. (**C**) UMAPs of individual histone modification profiles, of transcriptomes, and of all modalities combined. Gray lines connect modalities from the same single cell. Note: Only a subset of lines is shown on the basis of 100 randomly sampled cells. (**D**) ChromHMM-defined chromatin states (left) and alluvial plots to show changes in chromatin states over endoderm differentiation (right). Regions annotated as unmarked chromatin state at all time points are excluded from the alluvial plots. (**E**) Clustered smooth heatmaps show gene expression and associated histone modification levels at their promoters and gene bodies in metacells (*n* = 60) over differentiation pseudotime. Gene activities are scaled from 0 to 1 for each gene in each modality. (**F**) Line plots show transcriptional and histone modification dynamics for example genes in metacells (*n* = 60) along pseudotime. Gene activities are scaled from 0 to 1 for each gene in each modality.

We tested whether scMTR-seq could dissect chromatin state changes during lineage fate induction. We applied scMTR-seq to hPSC-derived definitive endoderm differentiation and profiled five histone modifications together with IgG and the transcriptome (Fig. 3A and table S2). Samples were collected at time points corresponding to undifferentiated cells (day 0), primitive streak (day 1), and definitive endoderm (days 2 and 3) (Fig. 3A). We obtained combined profiles of histone modifications and transcriptomes from 31,827 cells (44% recovery; fig. S7A). Transcriptome information yielded low collision rates of 0.68 to 0.86%, which is similar to rates reported in other combinatorial-barcoding methods (fig. S7B) (*13*, *22*).

Applying dimensionality reduction to the transcriptome data, followed by examination of marker genes, revealed clear separation of cell type by time point, indicating a synchronized exit of the undifferentiated state and induction of definitive endoderm identity (Fig. 3, A to C). This coordinated change in cell type was validated using flow cytometry analysis of lineage-specific markers (fig. S7C). As expected, dimensionality reduction using promoter-associated (H3K4me3 and H3K27me3) or gene body (H3K36me3) histone modification profiles showed relatively mild differences between cells from different time points (Fig. 3C). In contrast, profiles of enhancer-associated histone modifications (H3K4me1 and H3K27ac) showed greater change and distinguished major cell types (Fig. 3C). Notably, the ability of H3K4me1 and H3K27ac to separate cell types was dependent on sequencing depth and the size of genomic bins used in the analysis (fig. S7, D and E). Joint projection using all modalities also separated cell types into defined clusters (Fig. 3C and fig. S7, F to I).

We combined histone modification profiles from single cells at the same time point to generate aggregated profiles (Fig. 3B) followed by annotation of chromatin states by applying ChromHMM to the five histone modifications and IgG control datasets (Fig. 3, B and D; and fig. S8, A and B). This analysis revealed chromatin state changes over endoderm differentiation. For example, of 2231 bivalent promoters (H3K4me3 and H3K27me3) in undifferentiated cells, 44% maintained their bivalent status throughout differentiation, 19% transitioned to a repressed promoter state (H3K27me3 only), and 22% became activated (including endoderm-associated genes, such as *SOX17* and *GATA6*) (fig. S8C).

We next investigated the dynamics of histone modifications over endoderm differentiation using transcriptional pseudotime. We first created metacells by combining individual cells that were transcriptionally similar (fig. S8D) (*25*). Ordering metacells along pseudotime revealed patterns in the levels of histone modifications at gene promoters that correlated with the transcriptional activity of those genes (Fig. 3, E and F, and fig. S8E). For example, genes highly expressed in undifferentiated hPSCs, such as *POU5F1* and *SOX2*, showed progressive loss of active histone modifications over the time course (Fig. 3, E and F). Notably, there was no gain in H3K27me3 for these genes, suggesting that their transcriptional down-regulation is independent of this repressive modification. Genes associated with primitive streak, including *TBXT* and *WNT3*, were transcriptionally induced in a transient manner at day 1. This event was mirrored by dynamic changes in histone modifications at these gene loci including a transient gain in active modifications at day 1 and a decrease in H3K27me3 (Fig. 3, E and F). Last, *CXCR4* was transcriptionally up-regulated on day 2 of differentiation, and this is reflected by an increase in all of the profiled active histone modifications and by a decrease in the repressive modification H3K27me3 (Fig. 3, E and F, and fig. S8F). Together, these results demonstrate the application of scMTR-seq to resolve the dynamics of chromatin state change during lineage fate induction.

### Resolving lineage-specific chromatin state profiles in early mouse embryos

**Fig. 4.**
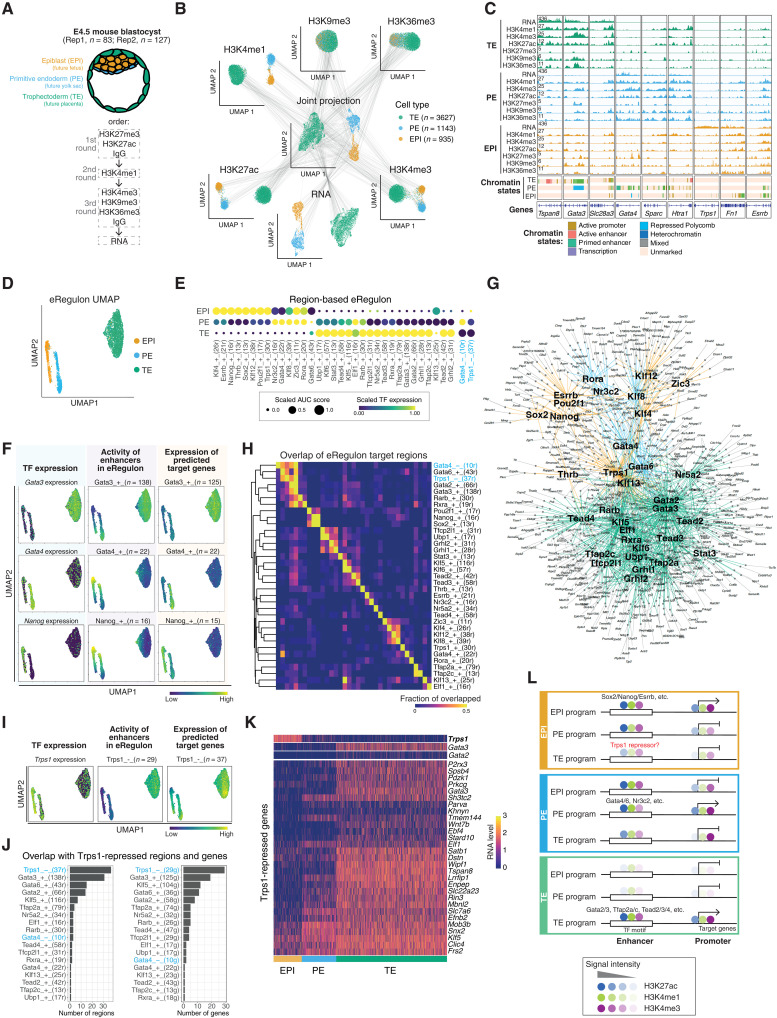
scMTR-seq reveals lineage-resolved gene regulatory patterns and networks in mouse embryos. (**A**) Schematic of blastocyst and order of sequential rounds of antibody tagmentation. (**B**) UMAPs of individual histone modification profiles, of transcriptomes, and of all modalities. Gray lines connect modalities from the same single cell. Only a subset of lines is shown on the basis of 100 randomly sampled cells. (**C**) Lineage-resolved histone modification and transcriptome profiles, and ChromHMM-defined chromatin states. (**D**) UMAP of 5705 blastocyst cells based on target gene and region enrichment scores of eRegulons. (**E**) Transcription factor expression of eRegulon on color scale, and activity of regions (based on H3K27ac) of eRegulon on size scale. +/− indicates whether transcription factor expression positively or negatively correlates with the expression of eRegulon target genes, respectively. Number of regions within each eRegulon in brackets. AUC, area under the curve. (**F**) UMAPs of three eRegulons (Gata3, Gata4, and Nanog) showing transcription factor expression of the eRegulon (left), activity of regions (based on H3K27ac) of the eRegulon (center), and expression of target genes of eRegulon (right). Values in center and right UMAPs are derived from AUCell enrichment scores. (**G**) Visualization of enhancer-driven gene regulatory networks in blastocysts. Nodes represent enhancers in individual eRegulons, connected to their predicted upstream transcription factors and to their inferred target genes. Edges colored by embryo lineage, assigned using lineage-specificity of transcription factor expression. (**H**) Overlap of target regions of eRegulons. The overlap is divided by number of target regions of the eRegulon in each row. (**I**) Same as (F) for Trps1(−) eRegulon. (**J**) Overlap of target regions (left) and target genes (right) between Trps1(−) eRegulon and all other eRegulons (top 18, ranked by overlap). (**K**) Expression of target genes in Trps1(−) eRegulon in individual cells, ordered by embryo lineage. (**L**) Lineage-specific histone modification patterns and examples of predicted transcription factors at gene regulatory regions.

Mouse blastocysts contain ~128 cells formed into three lineages: epiblast (EPI), trophectoderm (TE), and primitive endoderm (PE) (Fig. 4A). Low–cell number chromatin profiling methods have investigated the epigenomes of inner cells mass (containing a mixed population of EPI and PE) and TE (the outer layer of blastocysts) (*26*–*29*). However, these methods could not distinguish EPI from PE because they are not compatible with single-cell profiling, nor could they capture transcriptomes from the same cells to assign cell lineage. It is therefore unknown whether there are chromatin state differences between EPI and PE. To overcome this limitation, we dissociated embryonic day 4.5 (E4.5) mouse blastocysts into single cells and applied scMTR-seq to profile six different histone modifications plus IgG and the transcriptome in the same individual cells. To further improve the quality of the chromatin profiling libraries, we performed multiplexed antibody tagmentation in three sequential steps rather than all antibodies simultaneously (Fig. 4A and table S2). This adaptation led to a slight, but worthwhile, improvement in the specificity of histone modification profiles (fig. S9, A to D).

After filtering cells with sufficient DNA and RNA unique reads, a total of 5705 cells were retained in the final dataset, yielding an average of 27 cells per blastocyst (an estimated recovery of 21% cells, assuming that there were ~128 cells in each embryo). Transcriptome data identified three cell clusters corresponding to EPI (*n* = 935 cells), PE (*n* = 1143 cells), and TE (*n* = 3627 cells) (Fig. 4B and fig. S10, A to G). The relative proportions of cells corresponding to the three cell lineages are similar to intact E4.5 embryos, with a slight depletion of TE probably due to the resistance of this lineage to single-cell dissociation (*30*). Transcriptome data could also distinguish polar TE from mural TE (fig. S10, H and I). Dimensionality reduction of each histone modification individually revealed that H3K4me3, H3K4me1, H3K27ac, and H3K36me3 resolved cells into the three lineages, indicating that each lineage has a distinct epigenetic pattern (Fig. 4B and fig. S10, E and F). H3K9me3 could not resolve all lineages, which is in line with a prior study reporting that lineage-associated H3K9me3 programs become established in embryos after implantation (Fig. 4B and fig. S10, E and F) (*27*). H3K27me3 was too sparse to perform dimensionality reduction; this sparsity is consistent with the low levels of H3K27me3 previously reported in mouse blastocysts (*29*, *31*). Despite the sparsity of this modification, there were significantly more H3K27me3 reads in EPI compared to those in PE and TE (fig. S10J). This lineage-specific difference is in line with imaging-based data (*32*) and correlated with the higher expression levels of PRC2 components in EPI (fig. S10K).

To examine epigenome differences between the three embryo lineages, we computationally aggregated single-cell histone modification profiles for EPI, PE, and TE. Replicate datasets showed strong concordance (fig. S10L; average Pearson’s correlation coefficient between replicates was *r* = 0.85, with a range *r* = 0.64 to 0.99), and, for the few cases where comparisons were possible, histone modification patterns were broadly similar to published profiles of dissected inner cell mass and TE samples (fig. S10M). Leveraging our multiomic data of all three embryo lineages, we found that, although the promoters of EPI-associated genes were strongly marked by active histone modifications in EPI cells, the promoters of PE- and TE-associated genes unexpectedly carried relatively high levels of active modifications in EPI cells (Fig. 4C; fig. S11, A and B; and table S3). A similar pattern was observed for PE cells (Fig. 4C and fig. S11, A and B). In contrast, TE cells had a distinct pattern of histone modifications at promoters, whereby active histone modifications were only enriched at TE-specific promoters and were low or absent at EPI- and PE-associated gene promoters (Fig. 4C and fig. S11, A and B). Similar epigenetic asymmetries were detected for enhancers, whereby EPI- and PE-associated enhancers were marked by H3K27ac in EPI cells, but TE-associated enhancers were not (fig. S11C). In PE cells, enhancers associated with all three lineages had high or intermediate levels of H3K27ac, and, in TE cells, only TE-associated enhancers were marked by H3K27ac (fig. S11C).

We next used ChromHMM to integrate data from the six histone modification profiles and assign chromatin states (fig. S11D). Chromatin states associated with active regulatory regions and gene transcription matched the lineage-dependent patterns described above (Fig. 4C). In addition, in all embryo lineages, H3K9me3-defined heterochromatin was associated with repetitive elements (fig. S11E), and H3K27me3-associated repressive states were enriched at CpG islands (fig. S11E). Intriguingly, there was a weak enrichment of H3K27me3 in EPI at the promoters of PE-associated genes, as exemplified at the *Gata4* promoter (Fig. 4C and fig. S11B). This weak indication in EPI could signify the emergence of H3K27me3-marked regulatory regions, which is a signature that is strongly defined in E6.5 embryos (*31*). About two-thirds of genes marked by H3K27me3 in EPI at E4.5 are also marked by H3K27me3 in EPI at E6.5 (fig. S11, F and G).

These findings uncover asymmetrical lineage-specific patterns of chromatin states at gene regulatory regions. TE cells have active histone modifications only at TE-associated promoters and enhancers, whereas PE cells and, to a slightly lesser extent, EPI cells have active histone modifications at gene regulatory regions associated with all blastocyst lineages.

### Defining gene regulatory networks using histone modification profiles in embryo lineages

We next used the lineage-resolved histone modification and transcriptome profiles together with SCENIC+ (*33*) to predict enhancer-driven gene regulatory networks in early embryos (fig. S12A and tables S4 and S5). From this output, SCENIC+ defines eRegulons, which are groups of active enhancers that are predicted to be regulated by the same transcription factor and are associated with expression of enhancer-target genes. The expression of the transcription factor can either be positively or negatively correlated with the expression of the enhancer-target genes.

Dimensionality reduction based on eRegulon enrichment scores in single cells separates the three embryo lineages (Fig. 4D). Individual eRegulons included expected (e.g., Sox2 and Nanog for EPI, Gata6 and Nr3c2 for PE, and Tead4 and Gata3 for TE) and unanticipated (e.g., Thrb in EPI, Klf8 in PE, and Ubp1 in TE) transcription factor–centered networks (Fig. 4E and fig. S12, B and C). These connections are exemplified by Gata3, which is highly expressed in TE cells, and enhancers predicted to be targeted by Gata3 (*n* = 138 enhancers) have high activity specifically in TE cells (Fig. 4F). Furthermore, genes predicted to be targets of those enhancers (*n* = 125 genes) are highly expressed in TE cells and are positively correlated with *Gata3* expression in individual cells (Fig. 4F). Integrating the identified eRegulons visualizes enhancer-driven gene regulatory networks, highlighting the separation between EPI/PE and TE subnetworks, and shows the overlap in target enhancers and genes between factors (Fig. 4G). Clustering analysis also revealed associations and potential synergies between eRegulons (Fig. 4H and fig. S12D).

Examining the eRegulons revealed an interesting overlap in target enhancers and genes between Trps1 and the TE-specifying factors, Gata2 and Gata3 (Fig. 4, G and H). *Trps1* is highly expressed in EPI cells and is low in PE and TE (Fig. 4, E and I). Regions predicted to be repressed by Trps1 have low enhancer activity in EPI, whereas the same regions show high enhancer activity in PE and TE (where *Trps1* is not expressed; Fig. 4, E and I). Trps1 is an atypical Gata protein that binds to Gata motifs and functions as a repressor of other Gata factors (*34*, *35*), although a role for Trps1 has not been implicated before in blastocyst lineages. Many regions predicted to be repressed by Trps1 in EPI cells overlapped with regions defined as active enhancers in TE (Fig. 4I). In TE cells, these same enhancers are predicted to be downstream of Gata2/3 (Fig. 4J). Genes predicted to be negatively regulated by Trps1 are not transcribed in EPI, in line with a repressive role for Trps1 in EPI (Fig. 4K). In contrast, those genes are highly expressed in TE cells, where Trps1 is not present, but Gata2 and Gata3 are expressed and are predicted to positively regulate these genes (Fig. 4K). This set of genes is variably expressed in PE, which lack expression of *Trps1* and *Gata2/3* (Fig. 4K). Together, these findings suggest that Trsp1 might repress TE-promoting enhancer networks in EPI cells (Fig. 4L).

## DISCUSSION

Here, we introduce scMTR-seq, a method for combinatorial profiling of multiple histone modifications and transcriptome in single cells. scMTR-seq provides the ability to jointly capture multiple epigenome modalities, which is particularly valuable for identifying gene regulatory elements that require combinations of histone modifications to resolve, as well as for studying the interplay between different epigenetic features. This can also be achieved in heterogeneous samples or in rare cell types, due to the matched transcriptional profiles. The highly multiplexed nature of scMTR-seq also overcomes drawbacks of integrating single or low-number modalities into a combinatorial dataset.

Future development of scMTR-seq could include multiplexing additional antibodies. A challenge to achieve this stems from the variable capture efficiencies of different antibodies and enrichments of different targets in the cell, which can lead to uneven or poor sequence depth for some target modifications or proteins. Designing a target-specific enrichment strategy could be an option to overcome these technical challenges. Our findings also help to better understand the limitations of multitarget profiling methods, where some off-target signal is inevitable. We observe in scMTR-seq and in other multitarget methods that chromatin regions marked by high H3K27ac and H3K4me1 are most at risk from off-target effects. We reduce these effects in scMTR-seq by blocking free proteinA with IgG antibodies, by performing antibody-mediated tagmentation in sequential steps, and by normalizing histone modification profiles to IgG signal; efforts to further improve signal specificity and sensitivity remain a priority for the field. Last, innovations to increase the depth of sequence reads are also an area for future development, perhaps by implementing alternative methods for library amplification (*36*).

We used scMTR-seq to resolve lineage-specific chromatin maps of mouse embryos, which revealed differences in epigenetic patterns and gene regulatory networks between the three embryo lineages (Fig. 4L). These findings showed that TE cells have a distinct pattern of epigenetic modifications compared to EPI and PE cells, which might reflect their different lineage histories and the timing of lineage segregation events, as TE is the first lineage to be specified (*37*). Notably, we also found that promoters and enhancers associated with EPI, PE, and TE lineages had high levels of H3K27ac in PE cells; this may provide a molecular explanation underlying the lineage plasticity of PE cells (*38*). We further uncovered a predicted role for the transcription factor Trps1 in EPI cells as a repressor of TE-associated cis-regulatory networks. Our results predict a model where Trps1 and Gata2/3 target common regions but in different lineages, whereby Gata2/3 activates those regions and their target genes in TE cells, but Trsp1 represses them in EPI cells. Genes predicted to be downstream of Trps1 and Gata2/3 include *Gata3* itself (*39*, *40*), as well as other TE-associated factors, such as *Enpep* (*41*, *42*). Thus, Trps1 may function to suppress inappropriate expression of TE-associated genes and reinforce EPI-TE lineage segregation.

Together, scMTR-seq provides an incisive new technology to dissect chromatin-based mechanisms in a broad range of contexts. This could include small, heterogeneous samples, such as embryos, organoids and patient biopsies, or rare cell populations, such as blood progenitor cells. Last, although we focus our experiments on histone modifications, scMTR-seq is compatible with any antibody against proteins that bind chromatin and thus could also be used to examine combinations of transcription factor occupancy in single cells, aiding the discovery of gene regulatory mechanisms.

## MATERIALS AND METHODS

A detailed protocol for scMTR-seq is available from protocols.io (https://dx.doi.org/10.17504/protocols.io.yxmvmb7dog3p/v1). Information on the supplier and catalog numbers for reagents used are provided in table S1.

### Biological samples

#### Cell lines

WA09/H9 hPSCs were obtained from WiCell. The use of hPSCs for these experiments is approved by the Steering Committee of the UK Stem Cell Bank (SCSC11-58) and by the Human Research Committee at the Babraham Institute (HRP0021). Cells were grown in 5% CO_2_ and 5% O_2_ at 37°C and maintained in Essential 8 medium (Thermo Fisher Scientific) on 0.5% Geltrex (Thermo Fisher Scientific). Definitive endoderm was induced following the protocol described by Loh *et al.* (*43*), with modifications by Rostovskaya *et al.* (*44*). Specifically, undifferentiated hPSCs were seeded at a density of 15,000 cells/cm^2^ on Geltrex in Essential 8 medium. The next day, cells were washed with 0.1% bovine serum albumin (BSA) in Dulbecco’s modified Eagle’s medium and then cultured in endoderm induction medium comprising 50% Iscove’s modified Dulbecco’s medium (Thermo Fisher Scientific), 50% F-12 Nutrient Mix (Thermo Fisher Scientific), 1% Chemically Defined Lipids (Thermo Fisher Scientific), 0.1% BSA Fraction V (Thermo Fisher Scientific), transferrin (15 μg/ml; Merck), 450 μM monothioglycerol (Merck), insulin (0.7 μg/ml; Merck), activin-A (100 ng/ml; Cambridge Stem Cell Institute), 100 nM PI-103 (Tocris Bio-Techne), 3 μM CHIR99021 (Cambridge Stem Cell Institute), fibroblast growth factor 2 (FGF2; 10 ng/ml; Cambridge Stem Cell Institute), bone morphogenetic protein 4 (BMP4; 3 ng/ml; R&D Systems), and heparin (10 μg/ml; Merck). After 24 hours, cells were cultured in modified endoderm induction medium whereby CHIR99021 and BMP4 were removed, the concentration of FGF2 was increased to 20 ng/ml, and LDN193189 (Axon Medchem) was added to 250 nM.

#### Mouse embryos

Mice used in this study were bred and maintained in the Babraham Institute Biological Support Unit. Ambient temperature was ~19° to 21°C and relative humidity 52%. Lighting was provided on a 12-hour light:12-hour dark cycle including 15 min “dawn” and “dusk” periods of subdued lighting. After weaning, mice were transferred to individually ventilated cages with one to five mice per cage. Mice were fed CRM (P) VP diet (Special Diet Services) ad libitum and received seeds (e.g., sunflower and millet) at the time of cage cleaning as part of their environmental enrichment. All experimental procedures were performed under licenses issued by the Home Office (UK) in accordance with the Animals (Scientific Procedures) Act 1986 and were approved by the Animal Welfare and Ethical Review Body at the Babraham Institute. B6CBAF1 mice bred in-house were used experimentally between 8 and 12 weeks of age. Female mice were superovulated by injection with 5 IU of pregnant mare serum gonadotropin followed with 5 IU of human chorionic gonadotropin 46 hours later. Embryos at ~E3.5 were collected by flushing the uterus with M2 medium (Sigma-Aldrich) 94 to 98 hours after human chorionic gonadotropin injection. Embryos were cultured in potassium simplex optimized medium (Merck Millipore, MR-121-D) for 24 hours at 37°C in a 5% CO_2_ incubator under light mineral oil (Millipore, ES-005-C). If present, the zona pellucidae was removed from embryos using Acidic Tyrode’s solution (Sigma-Aldrich) and washed through three drops of M2 medium. Embryos were washed in PBSI [phosphate-buffered saline (PBS) without calcium or magnesium supplemented with 1× protease inhibitor cocktail (PIC), SUPERase·In ribonuclease (RNase) inhibitor (0.05 U/μl), Protector RNAse inhibitor (0.1 U/μl), and 0.2% BSA] and placed in 1.5-ml tubes containing PBSI. Embryos were incubated in TrypLE Express (Thermo Fisher Scientific) for 10 min at 37°C. Embryos were washed with PBSI and dissociated into single cells by pipetting. Nuclei were prepared as described in the “Sample preparation” section and stored at −80°C until use.

### Flow cytometry

Cells were dissociated by incubation with Accutase for 5 min at 37°C and washed with flow buffer (2% fetal bovine serum in PBS^−/−^). Cells were labeled with eFluor 780–conjugated Fixable Viability Dye (0.6 μl per test, detected using the 640-nm laser with 780/60 filters), BUV395-conjugated anti-CD24 antibody (1:40, BD Biosciences, clone ML5, detected using the 355-nm laser with 379/28 filters), allophycocyanin-conjugated anti-CD117 antibody (1:50, Thermo Fisher Scientific, CD11705, detected using the 640-nm laser with 670/14 filters), and phycoerythrin-conjugated anti-CXCR4 antibody (1:50, R&D Systems, FAB170B, detected using the 561-nm laser with 585/15 filters) for 30 min in the dark on ice. Cells were washed twice with flow buffer. Flow cytometry was performed at the Babraham Flow Core on a BD LSRFortessa and analyzed using FlowJo Analysis Software (FlowJo LLC).

### ProteinA Tn5 preparation and loading of MEDS

ProteinA-Tn5 expression plasmid (3XFlag-pA-Tn5-Fl, a gift from S. Henikoff; Addgene, plasmid no. 124601) (*45*) was transformed into T7 Express lysY/I q Competent *Escherichia coli* [New England Biolabs (NEB), C3013I]. A single colony was picked and propagated in 3 ml LB medium at 220 rpm, 37°C for 4 hours as a starter culture. The starter culture was added to 400 ml of LB with carbenicillin (100 μg/ml) and incubated at 220 rpm, 37°C until 0.6 optical density. The culture was cooled on ice for 30 min. Isopropyl-β-d-thiogalactopyranoside was added to the culture to a final concentration of 0.25 mM, and the bacteria were cultured overnight at 80 rpm and 18°C. Bacteria were collected by centrifugation at 10,000 rpm and 4°C for 30 min and resuspended in 40 ml of chilled HEGX buffer [20 mM Hepes-KOH (pH 7.2), 0.8 M NaCl, 1 mM EDTA, 10% glycerol, 0.2% Triton X-100, and 1× cOmplete PIC] and sonicated 10× for 45 s at 50% duty cycle while the tube was kept on ice. The supernatant of the lysed bacteria was mixed with 5 ml of chitin slurry (NEB, S6651S) and incubated on a rotator overnight at 4°C. The beads were rinsed with 20 ml of HEGX and mixed with 10 ml of HEGX plus 100 mM dithiothreitol (DTT) and incubated at 4°C for 48 hours with rotation to elute proteinA-Tn5. The elution was collected and dialyzed twice in Tn5 dialysis buffer [40 mM Hepes-KOH (pH 7.2), 100 mM NaCl, 0.1 mM EDTA, 0.85 mM DTT, 0.1% Triton X-100, and 10% glycerol] overnight at 4°C. ProteinA-Tn5 was concentrated with Ambicon Ultra-4 Centrifugal Filter Units 30k and mixed with an equal volume of sterile glycerol and stored at −20°C. ProteinA-Tn5 concentration was estimated by comparison to known BSA standards following Coomassie staining of SDS gels.

### Assemble proteinA-Tn5 with MEDS

All oligonucleotides (table S1) were purchased from Integrated DNA Technologies DNA and dissolved in STE buffer [10 mM tris (pH 8.0), 50 mM NaCl, and 1 mM EDTA]. To assemble proteinA-Tn5 with MEDS, 2 μl of 200 μM phosphorylated mosaic end adapter B dU (MEBdU) with different indexes [5 base pairs (bp)] or MEA was mixed with 2 μl of 200 μM blocked mosaic end–reverse and annealed using the following program: 95°C for 5 min, cooled down to 20°C with ramp rate of −1°C/min. Annealed adapters (4 μl) were mixed with 20 μl of proteinA-Tn5 (~5.5 μM) and incubated on a rotator at room temperature for 1 hour and then stored at −20°C for up to 1 year.

### Sample preparation

Samples were dissociated into single cells and washed twice with PBSI [Dulbecco’s phosphate-buffered saline without calcium or magnesium, 1× PIC, SUPERase·In RNase inhibitor (0.05 U/μl), Protector RNAse inhibitor (0.1 U/μl), and 0.04% BSA]. Cells were collected by centrifugation in a swing-out rotor at 300*g* for 3 min. Liquid was carefully aspirated, leaving ~20 μl remaining in the tube. Samples were mixed with the same volume of 2× Nuclei Extraction buffer [40 mM Hepes-KOH (pH 7.9), 20 mM KCl, 0.5 mM spermidine, 0.2% Triton X-100, 40% glycerol, 2× PIC, SUPERase·In RNase inhibitor (0.25 U/μl), and Protector RNAse inhibitor (0.5 U/μl)] and incubated on ice for 10 min. Nuclei were fixed with 0.2% formaldehyde at room temperature for 5 min and quenched with 1× Fixation Quench Buffer [5× Fixation Quench Buffer: 625 mM glycine, 250 mM tris-HCl (pH 8.0), 0.1% Triton X-100, and 0.5% BSA in Dulbecco’s phosphate-buffered saline without calcium or magnesium] on ice for 5 min. Nuclei were collected by centrifugation in a swing-out rotor at 4°C and 600*g* for 3 min and resuspended in 100 μl of Wash buffer [20 mM Hepes-NaOH (pH 7.5), 150 mM NaCl, 0.5 mM spermidine, 1% BSA, 1% PIC, SUPERase·In RNase inhibitor (0.125 U/μl), and Protector RNAse inhibitor (0.25 U/μl)]. Either nuclei were used fresh or, if they were to be frozen, DMSO was added to a final concentration of 10%, and samples were placed in a slow freezing container and stored at −80°C.

### In situ DNA tagmentation

To combine the selected antibody with the indexed proteinA-Tn5-MEBdU, 0.5 μl of antibody (1 μg/μl) and 0.5 μl of proteinA-Tn5 were mixed with 4 μl of Complete Wash 300 buffer [20 mM Hepes-NaOH (pH 7.5), 300 mM NaCl, 0.5 mM spermidine, 1% BSA, 1% PIC, SUPERase·In RNase inhibitor (0.125 U/μl), and Protector RNAse inhibitor (0.25 U/μl)] and incubated on an end-over-end rotator at room temperature for 1 hour. Rabbit IgG antibody (1 μl of 1 μg/μl) was added to each antibody-ProteinA-Tn5-MEBdU complex to block any uncombined proteinA-Tn5 and incubated at room temperature for 30 min. Blocked, preassembled antibody-proteinA-Tn5-MEBdU were pooled together and brought to a final volume of 100 μl with Complete Wash 300 buffer in the presence of 2 mM EDTA.

Preactivated paramagnetic concanavalin A (ConA; 20 to 100 μl) beads were added to the cell samples (either freshly prepared or thawed from frozen, as described above) and incubated at room temperature for 10 min. Nuclei bound to the ConA beads were washed with Wash buffer containing 2 mM EDTA and then incubated with blocked, preassembled antibody-proteinA-Tn5-MEBdU on a rotator at room temperature for 1 hour or overnight at 4°C. Cells were washed three times with modified Complete Wash 300 buffer [20 mM Hepes-NaOH (pH 7.5), 300 mM NaCl, 0.5 mM spermidine, 1% BSA, 1% PIC, SUPERase·In RNase inhibitor (0.05 U/μl), and Protector RNAse inhibitor (0.1 U/μl)]. Cells were suspended in 100 μl of Tag buffer [20 mM Hepes-NaOH (pH 7.5), 300 mM NaCl, 0.5 mM spermidine, 0.1% BSA, 1% PIC, SUPERase·In RNase inhibitor (0.25 U/μl), Protector RNAse inhibitor (0.5 U/μl), and 10 mM MgCl_2_] and incubated at 37°C for 1 hour. To stop tagmentation, samples were mixed with 3.34 μl of 0.5 M EDTA and 16 μl of 7.5% BSA and incubated on a rotator at room temperature for 5 min.

For sequential tagmentation, samples were washed twice with modified Complete Wash 300 buffer supplemented with 2 mM EDTA before incubated with preassembled antibody-proteinA-Tn5-MEBdU for the second round tagmentation. The same steps of DNA tagmentation were repeated as described above for the first round tagmentation. Further rounds of DNA tagmentation were repeated as required.

### In situ RT

Tagmented nuclei were washed twice with 100 μl of nuclei isolation buffer with RNase inhibitors (NIB-RI) buffer [10 mM Trizma buffer (pH 7.5), 10 mM NaCl, 3 mM MgCl_2_, 0.1% NP-40, 1% BSA, SUPERase·In RNase inhibitor (0.05 U/μl), Protector RNAse inhibitor (0.1 U/μl), and 1× PIC] and then resuspended in 50 μl of RT mix [1× RT buffer, 15% PEG-6000 (polyethylene glycol, molecular weight 6000), 10 μM RT primer, 0.5 mM deoxyribonucleotide triphosphate (dNTPs), SUPERase·In RNase inhibitor (0.5 U/μl), Protector RNAse inhibitor (0.5 U/μl), and Maxima H Minus RT (20 U/μl)]. RT was carried out by incubation at 50°C for 10 min and then three cycles of annealing (8°C, 12 s; 15°C, 45 s; 20°C, 45 s; 30°C, 30 s; 42°C, 120 s; and 50°C, 180 s), followed by 50°C for 5 min. Samples were diluted with 50 μl of NIB-RI buffer and washed once with 100 μl of NIB-RI buffer.

### Preparing oligonucleotides for single-cell barcoding

Linker oligonucleotides and barcode oligonucleotides for hybridization were prepared in RNase-free 96-well plates (twin.tec PCR plate, 96 LoBind, semi-skirted) with the combined oligonucleotides for each round of hybridization prepared in separate plates. Round 1 linker (final concentration, 9 μM) and BC1 barcodes (final concentration, 10 μM) were combined in a total volume of 10 μl per well in STE buffer [10 mM tris (pH 8.0), 50 mM NaCl, and 1 mM EDTA]. In a new plate, round two hybridization reagents combined 11 μM round 2 linker and 12 μM BC2 barcodes in 10 μl of STE buffer per well, and, in a new plate, round 3 hybridization reagents combined 13 μM round 3 linker and 14 μM BC3 barcodes in 10 μl of STE buffer per well. Oligonucleotides were annealed in a polymerase chain reaction (PCR) machine using the program: 95°C for 5 min, and cooling to 20°C at −1°C/min. Annealed oligonucleotides were stored at −20°C.

### Split-pool barcoding and ligation

Split-pool single-cell barcoding was performed on the basis of a previous study (*22*) with minor modifications. Samples after in situ RT were resuspended in 2 ml hybridization mix [1× T4 ligation buffer, SUPERase·In RNase inhibitor (0.05 U/μl), Protector RNAse inhibitor (0.32 U/μl), 1× PIC, 0.1% Triton X-100, 0.1% BSA, and 0.25× NIB]. Samples were then split and pooled for a total of three rounds with 48 barcodes in each round. Cells (40 μl) were aliquoted into each well of the round 1 plate (containing 10 μl of annealed round 1 linker and a unique BC1, as described above) and incubated at room temperature for 30 min in a thermomixer with agitation at 300 rpm. Then, 10 μl of blocker 1 solution (22 μM blocker 1 and 2× T4 ligation buffer) was added to each well of the round 1 plate and incubated at room temperature for 30 min in a thermomixer with agitation at 300 rpm. All cells in the plate were then combined and mixed. Cells (55 μl) were aliquoted into each well of the round 2 plate (containing round 2 linker and BC2), and the same procedure was followed as above, except that blocker 2 solution contained 26.4 μM blocker 2 and 2× T4 ligation buffer. Following this second round of barcoding and pooling, 70 μl of cells were aliquoted into each well of the round 3 plate (containing round 3 linker and BC3). Blocker 3 solution contained 23 μM blocker 3 and 0.1% Triton X-100. After Blocker 3 solution was added to the plate, and cells were combined and centrifuged at 4°C and 600*g* for 3 min. Nuclei were transferred into an Eppendorf protein LoBind tube and washed twice with 0.5 ml of NIB buffer. Nuclei were resuspended in 0.5 ml of ligation buffer [1× T4 ligation buffer, SUPERase·In RNase inhibitor (0.05 U/μl), Protector RNAse inhibitor (0.32 U/μl), 1× PIC, 0.1% Triton X-100, 0.1% BSA, 0.2× NIB, and T4 DNA ligase (20 U/μl)] and incubated at 25°C for 30 min in a thermomixer with agitation at 300 rpm. The tube containing the nuclei was attached to a magnetic rack, and the nuclei were washed with 0.5 ml of NIB and then resuspended in 0.5 ml of NIB and passed through a 30-μm strainer. The nuclei were counted with a hemocytometer and aliquoted into multiple tubes to generate sublibraries, with a defined number of cells per sublibrary based on the estimated collision rate (RT/MEBdU × BC1 × BC2 × BC3).

### Reverse cross-linking and lysis

The volume of each sublibrary was brought to 20 μl with NIB buffer. Twenty microliters of 2× reverse cross-linking buffer [100 mM tris (pH 8.0), 100 mM NaCl, 0.04% SDS, 2 μl of proteinase K (20 mg/ml), 1.6 μl of RI mix (SUPERase RI:Protector RI 1:1)] were mixed with each sample and incubated at 55°C for 1 hour in a PCR machine. Phenylmethylsulfonyl fluoride (2.5 μl of 100 mM) was added to the reverse cross-linked sample to inactivate proteinase K and incubated at room temperature for 10 min. Samples were attached to a magnetic rack, and the supernatant was transferred into a new tube, leaving the ConA beads behind.

### Separation of cDNA from gDNA

MyOne C1 Streptavidin Dynabeads (10 μl) were washed with 800 μl of 1× B&W-T buffer three times and resuspended in 45 μl of 2× B&W buffer with 1 μl of SUPERase RI. Prepared C1 beads were mixed with lysed, reverse cross-linked samples and incubated on an end-to-end rotator at 10 rpm for 60 min at room temperature to bind the biotin-labeled cDNA. The tubes were then attached to a magnetic rack to separate tagmented genomic DNA (gDNA; in supernatant) from cDNA (attached to the beads).

### DNA library amplification

For DNA library preparation, tagmented gDNA was purified with 1.2× volume of Ampure XP beads and eluted with 21 μl of 0.1× elution buffer (EB). For adapter switching and amplification, we followed the procedure as described previously (*20*) with minor modifications. Eight microliters of 2× NEBNext PCR master mix was combined with 20 μl of eluted sample from the supernatant and incubated at 72°C for 10 min in a PCR machine. Three microliters of 1 μM MEALNA was added to the mixture to perform adapter switching with the following program: 98°C for 30 s; 10 cycles of 98°C for 10 s, 59°C for 20 s, and 72°C for 10 s. After this step, 2 μl of 25 μM indexed P7, 2 μl of 25 μM indexed Ad1, 50 μl of Q5U master mix, and 15 μl of H_2_O were added, and the libraries were amplified with the following program: 98°C for 30 s; 16 cycles of 98°C for 10 s, 55°C for 20 s, and 72°C for 30 s; and 72°C for 3 min. PCR products were purified with 0.7× Ampure XP beads and eluted with 32 μl of EB. The concentration of libraries was measured with a Qubit. Samples were run on a bioanalyzer to check the size distribution of library fragments. Libraries were sequenced on Illumina NovaSeq 6000, NextSeq 500, or Element Biosciences AVITI instruments at the Babraham Institute Genomics Facility and the CRUK Cambridge Institute Genomics Facility.

### RNA library preparation

#### Template switching

For RNA library preparation, we followed the procedure described previously (*22*) with minor modifications. cDNA bound to the C1 beads was washed three times with 100 μl of 1× B&W-T buffer containing SUPERase inhibitor and once with 100 μl of STE buffer containing SUPERase inhibitor. Beads were then resuspended in 50 μl of template switching mix (10 μl of 5× RT buffer, 10 μl of 20% Ficoll PM-400, 15 μl of 50% PEG-6000, 1.25 μl of 100 μM template-switching oligonucleotide, 2 μl of 25 mM dNTPs, 0.625 μl of Protector RNase inhibitor, 0.625 μl or SUPERase RNase inhibitor, 2.5 μl of Maxima H Minus RT (200 U/μl), and 8 μl H_2_O). The reaction was incubated on an end-to-end rotator at 10 rpm for 30 min at room temperature and then incubated for 90 min at 42°C in a PCR machine with mixing every 30 min.

#### Preamplification

After template switching, the cDNA-bound C1 beads were diluted with 100 μl of STE and washed with 200 μl of STE without disturbing the bead pellet. Then, the beads were resuspended in 50 μl preamplification mix (25 μl of Kapa HiFi PCR mix, 2 μl of 10 μM preamplification forward primer, 2 μl of 10 μM preamplification reverse primer, and 21 μl of H_2_O) and cycled in a PCR machine using following program: 95°C for 3 min; 11 cycles of 98°C for 30 s, 65°C for 45 s, and 72°C for 3 min; then 72°C for 5 min. PCR products were purified by 0.8× AMPure XP beads and eluted to 32 μl of 0.1× EB. The concentration of libraries was measured with a Qubit. Samples were run on a Bioanalyzer to check the size distribution of library fragments.

#### Tagging MEA and PCR amplification

MEA adapters were next added to the preamplified RNA library. Libraries (50 ng) were mixed with 2 μl of 1:20 diluted blocked Tn5-MEAin 5 μl of 4× Tagmentation buffer, and H_2_O was added to a final volume of 20 μl. Samples were incubated at 37°C for 30 min. Two microliters of 0.2% SDS were added to the reaction and incubated at room temperature for 10 min to release tagged fragments. One microliter of 4% Triton X-100 was added to neutralize the SDS before being mixed with 25 μl of 2× NEBnext PCR master mix, 1 μl of 25 μM indexed Ad1, and 1 μl of 25 μM indexed P7. PCR was performed with the following program: 72°C for 10 min and 98°C for 3 min; 11 cycles of 98°C for 10 s, 65°C for 30 s, and 72°C for 1 min; and 72°C for 5 min. PCR products were purified using 0.7× AMpure beads and eluted with 32 μl of EB. The concentration of libraries was measured with a Qubit. Samples were run on a bioanalyzer to check the size distribution of library fragments. Libraries were sequenced on Illumina NovaSeq 6000, NextSeq 500, or Element Biosciences AVITI instruments at the Babraham Institute Genomics Facility and the CRUK Cambridge Institute Genomics Facility.

### Data processing

#### Processing scMTR-seq data

After sequencing, cell barcodes and unique molecular identifier (UMI) information were extracted with modified Reachtools (*13*). Sequences from fixed positions of Read2 were extracted: 1st to 8th for BC3, 39th to 46th for BC2, 77th to 84th for BC1, 115th to 119th for RT-ID in RNA data or for antibody-ID in DNA data, and 120th to 127th for UMI. Barcode combinations (BC1:BC2:BC3:RT-ID/antibody-ID) were mapped to all possible barcode combinations with Bowtie (*46*) (version 1.3.1, --norc -m 1 -v 0). Mapped cell IDs and UMI were stored in read names of genome sequences. Trim-galore (version 0.6.10) was used to trim adapter sequences with default settings. Trimmed reads were mapped to GRCh38 for human samples and mm10 for mouse samples using Bowtie2 (*47*) (version 2.5.3) for DNA data and STAR (*48*) (version 2.7.11b) for RNA data with default settings. Only unique mapped reads were kept by samtools filtering (version 1.19.2, mapping quality > 50 for RNA data and mapping quality > 10 for DNA data). For the paired-end mapping data, only proper mapped reads with correct orientation were kept. Duplicated reads were identified by the mapping positions and UMIs for each cell ID of RNA data. Duplicated reads were identified by mapping positions only for each cell ID of DNA data. RNA deduplicated data were feature counted by custom script with splitpoolquantitation and converted to sparse matrix. DNA deduplicated data were converted to fragment files for downstream analyses.

### Peak calling

SEACR (*49*) (version 1.3) was used for de novo peak calling with parameters: -c 0.02 -n norm -m stringent for samples without IgG control or parameters: -n norm -m stringent for samples with IgG control.

### Profiling of histone modification signals

deepTools (*50*) (version 3.5.4) was used to generate bigWig files with bamCoverage (--binSize 50 --normalizeUsing RPGC --ignoreForNormalization chrX --extendReads 200) and bigwigCompare (--operation ratio --binSize 50 --pseudocount 1) functions. BigWig tracks were visualized in genome browser with IGV tools (*51*) (version 2.18.0). Average signals of histone modifications over sets of ENCODE ChIP-seq peaks, transcription start site (TSS) regions or enhancer regions were generated by ComputMatrix (reference-point -a 5000 -b 5000 --referencePoint center) and plotProfile and plotHeatmap (--zMin -3 --zMax 3) functions from deepTools.

### Clustering and dimensional reduction

RNA matrix and DNA genome-wide bin matrix with 5-kb nonoverlapping bins or other length as specified from single-cell datasets were loaded to Seurat (*52*) (version 5.1.0) and Signac (*53*) (version 1.13.0) for clustering, marker identification, and dimensional reduction. For RNA data, the top 2000 variable genes were selected with FindVariableFeatures and used for principal components analysis (PCA) with RunPCA function in Seurat. All genes were used for RNA data normalization with ScaleData function. For clustering analyses, PCA dimensions (1:10 for hPSC differentiation datasets, 1:15 for mouse E4.5 blastocyst datasets) were used for building the shared nearest neighbor (SNN) graph with FindNeighbors function and followed to identify clusters with original Louvain algorithm using FindClusters functions. Two-dimensional visualizations used Uniform Manifold Approximation and Projection (UMAP) with PCA dimensions (1:10 for hPSC differentiation datasets, 1:15 for mouse E4.5 blastocyst datasets) by RunUMAP function. FindAllMarkers was used to identify markers between clusters. For DNA data, Genome Bin Matrix was normalized with median counts of histone modification fragments of single cells for each histone modification followed by latent semantic indexing (LSI) using RunTFIDF and RunSVD functions in Signac. UMAP was used for two-dimensional visualizations by RunUMAP using LSI dimensions 2:10 for hPSC differentiation datasets and 2:30 for mouse E4.5 blastocyst datasets (the first dimension is normally highly associated with sequencing depth). For clustering analyses, *k*-nearest neighbors and SNN graph were calculated using FindNeighbors, followed by smart local moving (SLM) community detection algorithm to determine clusters with FindClusters function in Seurat. Weighted nearest neighbor (WNN) analysis for hPSC differentiation datasets was performed with reduction.list = (“pca.RNA,” “lsi.H3K27ac,” “lsi.H3K27me3,” “lsi.H3K4me1,” “lsi.H3K4me3,” and “lsi.H3K36me3”) and dims = list (1:10, 2:10, 2:10, 2:10, 2:10, and 2:10) with FindMultiModalNeighbors function in Seurat. For mouse E4.5 blastocyst, WNN analysis was performed with reduction.list = (“pca.RNA,” “lsi.H3K27ac,” “lsi.H3K9me3,” “lsi.H3K4me1,” “lsi.H3K4me3,” and “lsi.H3K36me3”) and dims = list (1:15, 2:30, 2:30, 2:30, 2:30, and 2:30) with FindMultiModalNeighbors function in Seurat. Cell clustering was determined by using the WNN graph with SLM algorithm by running the FindClusters function in Seurat with resolution = 0.1. To quantify the accuracy of cell lineage separation based on histone modification data with different sequencing depths, we calculated the fraction of *k*-nearest neighbors (*k* = 50) for each cell that belonged to the same cell-type classification based on the RNA data.

### Chromatin states identification

ChromHMM (*24*) (version 1.25) was used for chromatin states analyses with bulk data or aggregated single-cell datasets. For hPSC differentiation data, reads in 200-bp bins were binarized with BinarizedBed and poissonthreshold -p 5e-7, using IgG as a control. For E4.5 mouse blastocyst, reads in 1000 bp bins were binarized with BinarizedBed and poissonthreshold -p 5e-7. Total of 15 chromatin states were specified for Learnmodel. MakeSegmentation was used to sign chromatin states for downsampled data. Chromatin states were annotated on the basis of the emission probability of combined histone modifications and the distance to TSSs. Target genes of chromatin states were annotated by annotatePeaks from Homer (version 4.11.1), with cutoff of the distance to TSS within 5 kb. To track the changes of bivalent promoters in hPSCs during endoderm differentiation, we first define bivalent marked genes in hPSCs by checking the ratio of chromatin states within the gene TSS upstream 1000 bp and downstream 1000 bp. The highest representing chromatin states in the promoter at different time points were assigned to the gene. Chromatin states were ordered from day 3 to day 0 to track the changes during endoderm differentiation and visualized in heatmap plotted by pheatmap package in R.

### Metacells

We identified metacells (groups of cells that represent singular cell states from single-cell data) with the goal of achieving a resolution that retains the continuous nature of differentiation trajectories while overcoming the sparsity issues of single-cell data. We adopted the SEACells (*25*) (version 0.3.0) implementation. The SEACells parameter was set to 60 to adjust the average H3K27ac reads to ~1 million, as previously recommended (*25*). The SEACells model was fitted using n_waypoint_eigs = 10 and convergence_epsilon = 1e-5, with kernel built on PCA.

Expression levels of metacells were generated by AggregateExpression function in Seurat. Protein coding genes were kept for downstream analysis. The top 2000 variable genes were selected with FindVariableFeatures and used for PCA with RunPCA function in Seurat. PC1 was ranked and used as pseudotime order of metacells. For clustering analyses, PCA dimensions 1:20 were used for building the SNN graph with FindNeighbors function and followed to identify clusters with original Louvain algorithm using FindClusters functions with resolution = 2. For metacell DNA data, genome-wide nonoverlapping 5-kb bin matrices were generated by GenomeBinMatrix function in Signac. Gene activities were calculated by GeneActivity function in Signac with default setting for each histone modification. Gene activities were normalized with LogNormalize method and scaled with the average fragment counts of each histone modification. For visualization of the coordinated changes in different modalities of individual marker genes along pseudotime, we plotted line plots with geom_smooth (method = “loess,” formula = “*y* ~ *x*”) function in ggplot2. For the heatmaps plot, we chose the top 300 marker genes ordered by absolute fold changes of expression level from each cluster of RNA data. Genes were unsupervised clustered using hclust function with the ward.D2 method. Metacell data matrix was further smoothed with average five closest metacells along pseudotime for each gene and visualized with pheatmap.

### Gene regulatory networks

#### Identification of enhancers with shared patterns

For topic modeling, we used pycisTopic (*54*) (version 1.0a0) on H3K27ac dataset. First, we generated pseudobulks for each cell type and performed peak calling using MACS2 (*55*) (version 2.2.7.1) with the parameters --format BEDPE --keep-dup all --shift 73 --ext_size 146. To obtain a consensus set of peaks, we applied the iterative overlap peak merging method as outlined previously (*54*). In this method, each summit is extended by a peak_half_width (default, 250 bp) in both directions, and we progressively filter out peaks with a higher Benjamini-Hochberg adjusted *P* value that overlap with peaks that have a lower adjusted *P* value. Depending on the number of peaks in a merged region, the following occurs: For a single peak, the original peak is retained; for two peaks, the peak with the lower adjusted *P* value is kept; and for three or more peaks, the region with the lowest adjusted *P* value is selected and any overlapping original peaks are removed. This process is repeated for the peak with the next lowest adjusted *P* value until all peaks are processed. We then carried out topic modeling using 500 iterations and an eta of 0.1, exploring between two topics and a range from 5 to 10, as we identified that the optimal model fell within this range based on the model selection metrics and the dimensionality reduction results. In addition, we ultimately selected a model with 10 topics. Dropout imputation was carried out by multiplying region-topic and topic-cell probabilities. The imputed histone modification matrix was scaled by 10^6^. Regions differentially enriched with H3K27ac were identified across all cell populations using default settings, and topics were binarized via Otsu thresholding (*54*).

#### Motif enrichment analysis

To identify enriched motifs and infer transcription factor (TF) cistromes (that is, sets of regions in which a TF motif is present), pycisTarget (*33*) (version 1.0.3.dev2+g81eb875) was run with and without promoters, species as *Mus musculus*. Specifically, we used regions differentially enriched with H3K27ac in each cell type and binarized topics (with Otsu thresholding), overlapped with enhancers regions identified by chromHMM, to make full use of chromatin states inferred from all the histone modifications that we profiled. The rankings, score, and motif annotation databases used are available on https://resources.aertslab.org/cistarget/.

#### Prediction of enhancer-driven gene regulatory networks

TF cistromes identified through motif enrichment analysis with pycisTarget were used as input for SCENIC+ (*33*) (version 1.0.1.dev8+g40ffe5a). SCENIC+ was executed with default parameters, using http://sep2019.archive.ensembl.org as the Biomart host. The search space for region-to-gene relationships was defined as the maximum of either the boundary of the nearest gene or 150 kb, and the minimum was set to 1 kb upstream of the TSS or downstream of the gene’s end. TF-to-gene relationships were inferred using gradient boosting machine regression between all TFs and genes. Final eRegulons were constructed via a gene set enrichment analysis–based approach. In this, region-to-gene relationships were binarized on the basis of gradient boosting machine regression importance scores, with binarization carried out using the BASC (*56*) method. We further refined the results by retaining only eRegulons with extended annotations when no direct annotation was available, as the confidence level of direct motif annotations is generally higher. Additionally, eRegulons with a negative region-to-gene correlation were discarded, as they are often considered noisy. The gene-based and region-based eRegulon enrichment scores were then determined using AUCell (*56*) (version 0.1.2.dev2+g1ffcf0f) with threshold of 0.05. The network of eRegulons was plotted by Cytoscape (*57*).
