# Supplementary material for: Combinatorial profiling of multiple histone modifications and transcriptome in single cells using scMTR-seq
Source: Sci Adv. 2025 Aug 6;11(32):eadu3308. doi: 10.1126/sciadv.adu3308 (PMC12327464; doi:10.1126/sciadv.adu3308)
Supplement: Supplementary file 1 — Figs. S1 to S12 Legends for tables S1 to S5 [file sciadv.adu3308_sm.pdf]

Science Advances
AAAS

Supplementary Materials for

Combinatorial profiling of multiple histone modifications and transcriptome in single cells using scMTR-seq

Yang Wang *et al.*

Corresponding author: Peter J. Rugg-Gunn, peter.rugg-gunn@babraham.ac.uk

Sci. Adv. **11**, eadu3308 (2025)
DOI: 10.1126/sciadv.adu3308

The PDF file includes:

Figs. S1 to S12
Legends for tables S1 to S5

Other Supplementary Material for this manuscript includes the following:

Tables S1 to S5

Figure S1

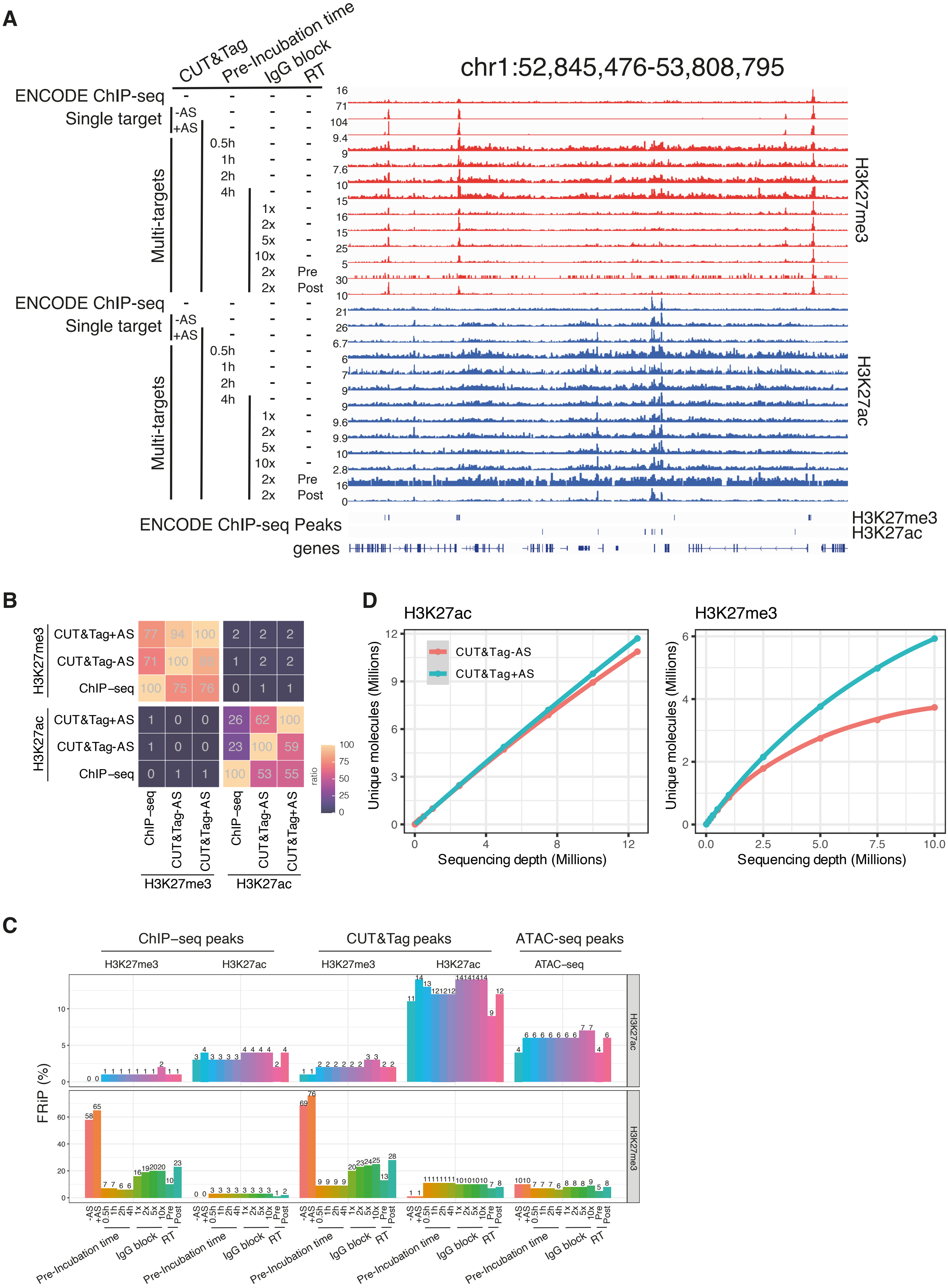
A
CUT&Tag
Pre-Incubation time
IgG block
RT
chr1:52,845,476-53,808,795
ENCODE ChIP-seq
Single target
-AS
+AS
Multi-targets
0.5h
1h
2h
4h
1x
2x
5x
10x
Pre
Post
H3K27me3
H3K27ac
ENCODE ChIP-seq Peaks
genes
B
CUT&Tag+AS
CUT&Tag-AS
ChIP–seq
ratio
D
H3K27ac
H3K27me3
CUT&Tag-AS
CUT&Tag+AS
Unique molecules (Millions)
Sequencing depth (Millions)
C
ChIP–seq peaks
CUT&Tag peaks
ATAC-seq peaks
ATAC–seq
FRiP (%)
Pre-Incubation time
IgG block
RT

Fig. S1. Adapter switching achieves higher CUT&Tag library complexity.

(**A**), Genome browser tracks show H3K27me3 (red) and H3K27ac (blue) signals in hPSCs under various conditions tested. Tracks compare ENCODE ChIP-seq, single modality CUT&Tag and our multi-modality method (H3K27me3 and H3K27ac, with and without joint transcriptome profiling). AS, adapter switching. Pre-incubation time refers to the duration when the antibody-proteinA-Tn5-adapter complexes were combined. IgG block refers to the amount of IgG antibody used to block any unreacted protein A-Tn5. RT, reverse transcription step, where "pre" means transcriptome profiling before the Tn5 tagmentation step, and "post" means after Tn5 tagmentation.

(**B**), Heatmaps show the percentage overlap of H3K27me3 and H3K27ac peaks between ENCODE ChIP-seq and CUT&Tag with or without adapter switching.

(**C**), Fraction of reads in peaks (FRiP) as a measure of on-target specificity. Peaks were defined using ENCODE ChIP-seq, single-target CUT&Tag or ATAC-seq data as indicated.

(**D**), Plots show that implementing adapter switching in CUT&Tag leads to the recovery of more unique reads with different downsampled sequencing depth.

Figure S2

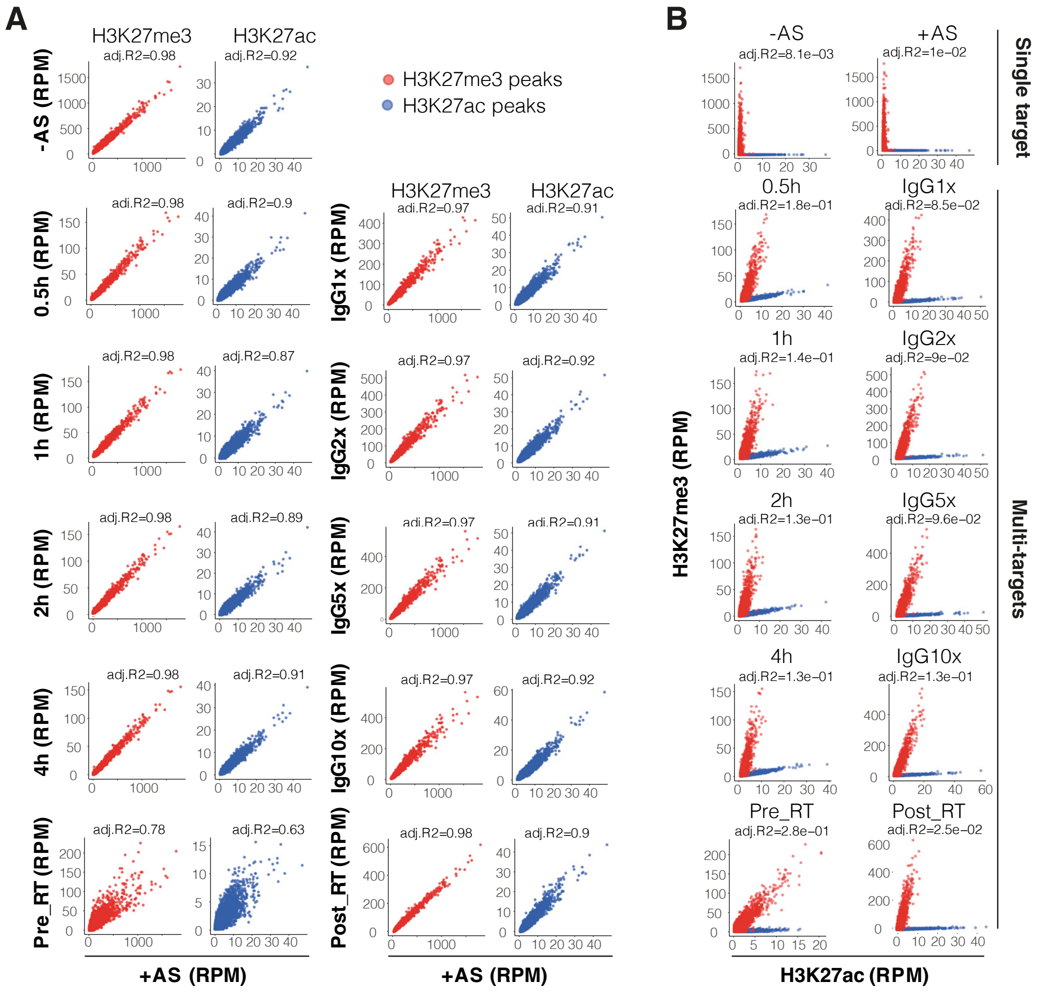

C

Multi-Targets

Single Target		H3K27me3: Pre-incubation time 0.5h	1h	2h	4h	IgG block 1x	2x	5x	10x	RT Pre	Post	H3K27ac: Pre-incubation time 0.5h	1h	2h	4h	IgG block 1x	2x	5x	10x	RT Pre	Post
H3K27me3	CUT&Tag+AS	69	66	68	73	75	76	74	81	39	94	13	10	9	8	14	17	15	20	7	12
	CUT&Tag-AS	64	61	63	68	69	70	69	75	35	89	12	9	8	8	12	16	14	18	6	11
	ChIP–seq	45	39	43	49	48	49	47	53	22	71	4	3	2	3	5	6	6	9	3	6
H3K27ac	CUT&Tag+AS	38	28	32	40	13	11	9	12	1	12	51	42	47	53	49	53	51	54	24	53
	CUT&Tag-AS	33	25	28	35	12	10	8	11	1	11	45	37	41	46	43	47	45	47	25	49
	ChIP–seq	28	24	26	30	14	12	10	13	0	17	38	34	37	39	42	46	42	45	16	59

ratio: 0, 25, 50, 75, 100

Fig. S2. Optimisation of method though adapter switching and IgG blocking antibodies.

(**A**), Scatter plots show on-target signals (reads per million, RPM) detected between different assays in peaks defined by ENCODE ChIP-seq datasets. X-axes show single target assays with adapter switching; y-axes show different conditions including single target assays without adapter switching, multi-targets assays with varying durations when the antibody-proteinA-Tn5-adapter complexes were combined, with increasing amounts of IgG blocking antibody, and with transcriptome profiling before/after the Tn5 tagmentation step. Coefficients of determination are shown (adjusted R2).

(**B**), Scatter plots show mutually exclusive signal of H3K27me3 and H3K27ac detected in single target assays with adapter switching (+AS) or not (-AS) and in multi-targets assays with different conditions. X-axes show H3K27ac signal; y-axes show H3K27me3 signal. Peaks used are defined by ENCODE ChIP-seq datasets.

(**C**), Heatmaps show the percentage overlap between peaks defined using datasets of ENCODE ChIP-seq or single-target CUT&Tag with adapter switching (+AS) or not (-AS) and multi-targets assays with different conditions. The overlap is divided by the number of peaks of the data in each row.

Figure S3

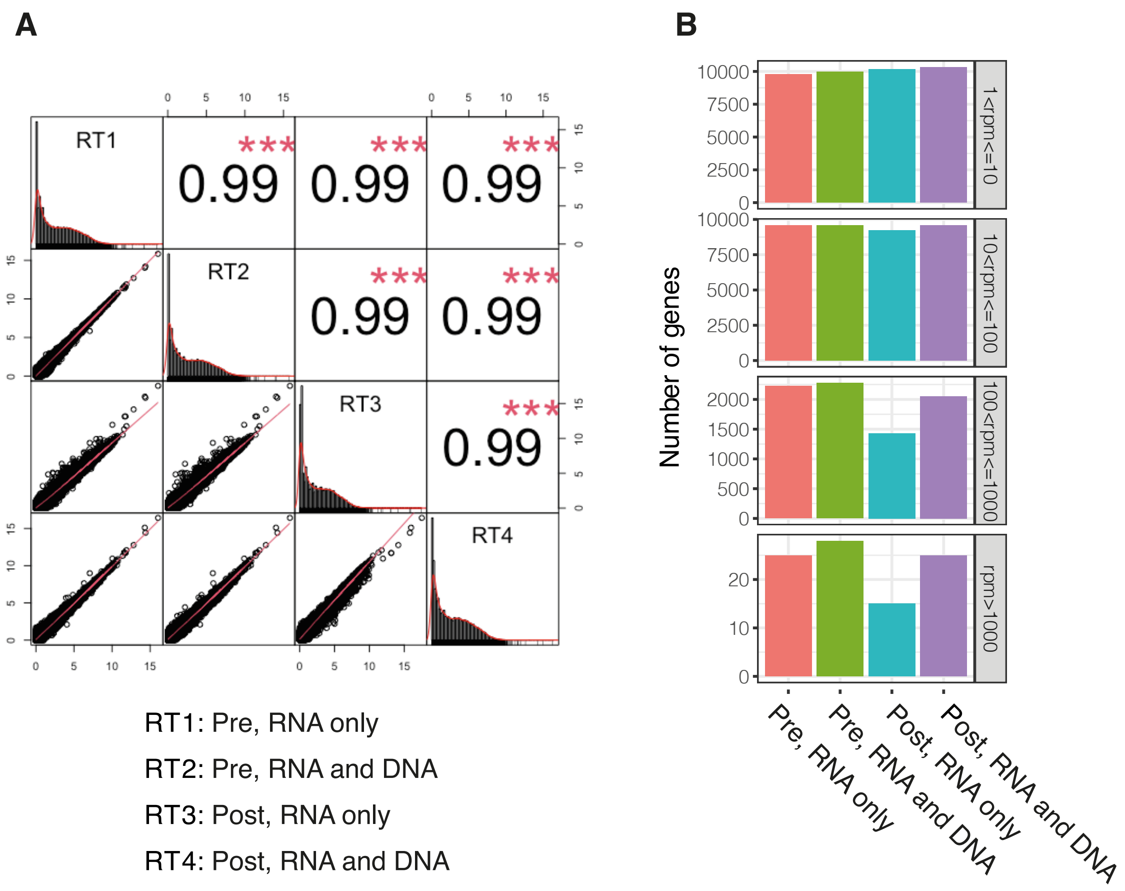

RT1: Pre, RNA only

RT2: Pre, RNA and DNA

RT3: Post, RNA only

RT4: Post, RNA and DNA

Fig. S3. Optimisation of transcriptional profiling.

(**A**), Strong correlation between transcriptomes that were profiled either pre- or post-Tn5 tagmentation.

(**B**), Number of genes obtained through transcription profiling with the indicated conditions.

Figure S4

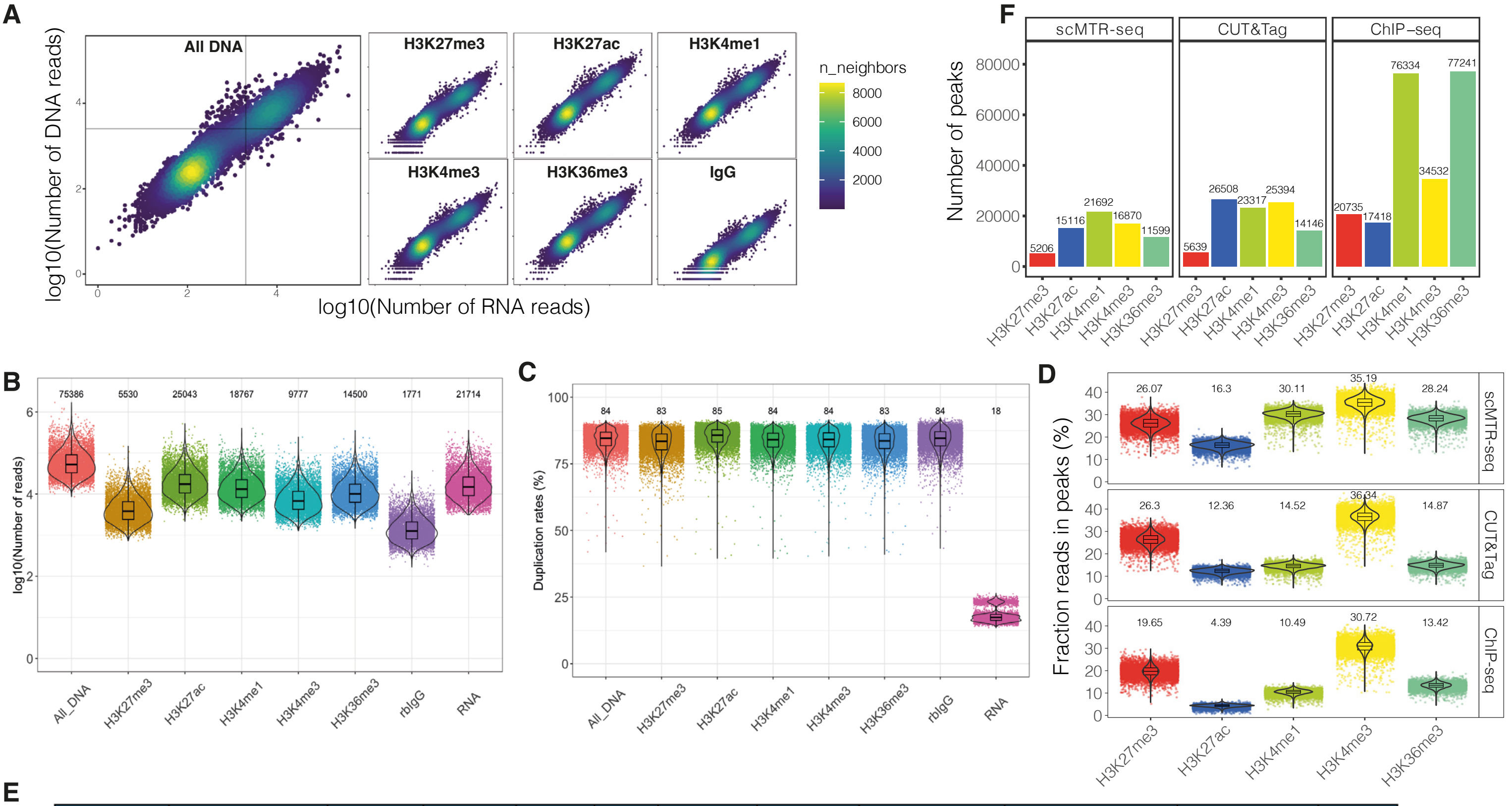

E

Method	Add multiplex	Co-tagment	Seq-tagment	pA-Tn5 blocking	RNA	Number of multiplex	DNA reads per cell	RNA reads per cell (UMIs/Genes)	Throughput	Starting cell number	Recovery rate
Multi-CUT&Tag	antibody conjugation	+	-	-	-	3	1000	-	High10x	200k	31.2
Nano-CT	Nanobody	+	-	-	-	3	22000	-	High/10x	200k	28-68%
NTT-seq	Nanobody	+	-	-	-	3	1400	-	High/10x	1M	-
MulTi-Tag	antibody conjugation	-	+	-	-	3	3500	-	Medium/iCell nanowell	510k	4%
uCoTarget	preincubation	-	+	-	-	5	11000	-	Very high/Split-pool	300k-1M*	-
uCoTargetX	preincubation	-	+	-	+	2	24000	1691/1366	Very high/Split-pool	300k-1M*	-
MAbID	antibody conjugation	+	-	-	-	6	2500	-	Low/384 plates	750k	57.70%
scMTR-seq	preincubation	+	+	+	+	6	10000	11827/2559	Very high/Split-pool	10k-100k	40%

Note: * estimated based on the requirement of 3,000-10,000 cells per well x 96 well in split-pool procedure.

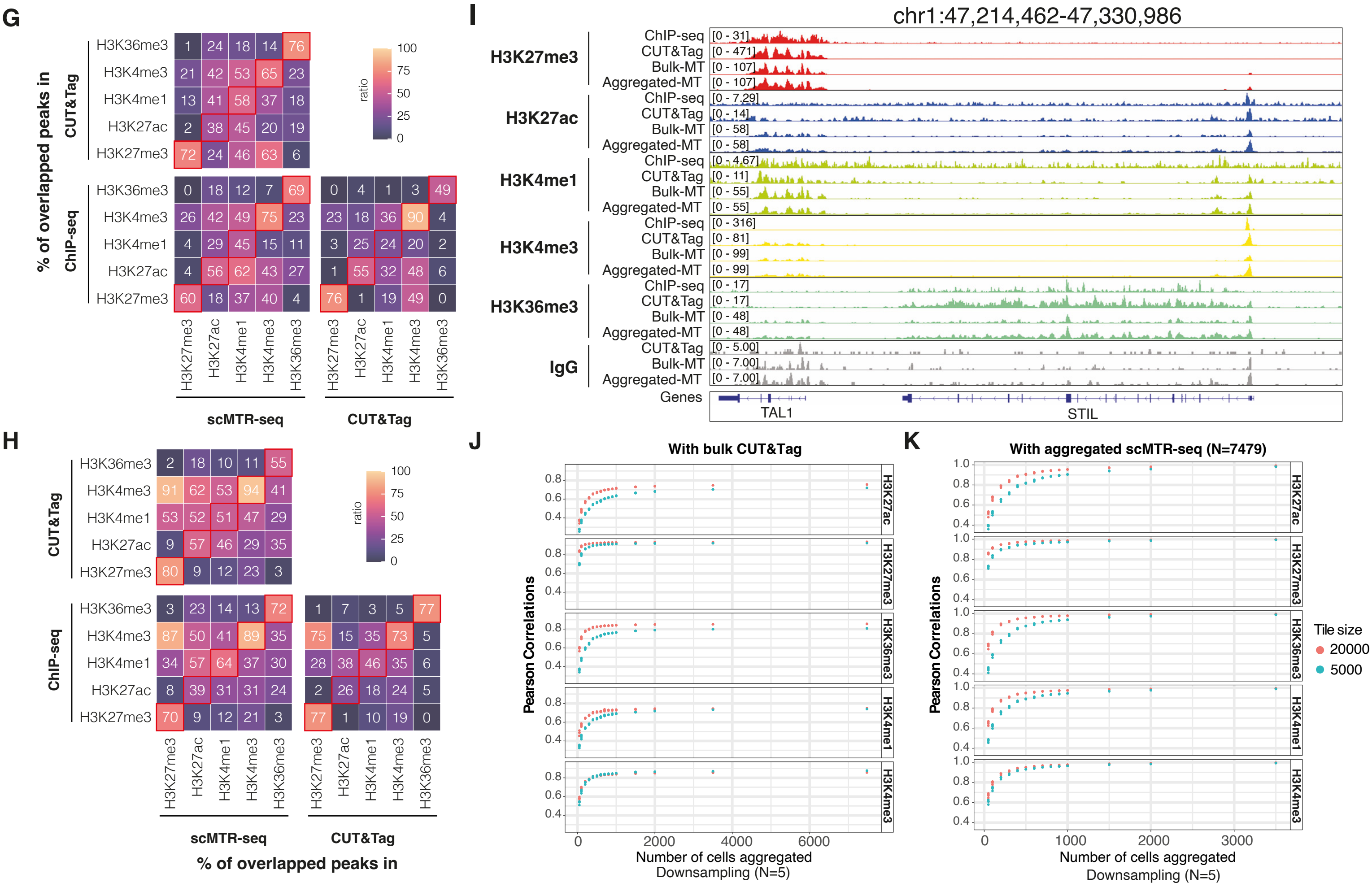

Fig. S4. Quality control information for scMTR-seq.

(**A**), Density plots show the distribution of number of RNA reads (x-axes) and the number of DNA reads (y-axes, for each histone modification individually, or all total reads) detected per cell.

(**B**), Violin plot of unique reads per cell for indicated modality. Numbers above each dataset shows mean values. Boxplots show median with interquartile range and minimum/maximum whiskers. Dot plots show the unique reads per cell for indicated modality.

(**C**), Violin plot of duplication rates per cell for indicated modality. Numbers above each dataset shows mean values. Boxplots show median with interquartile range and minimum/maximum whiskers. Dot plots show the duplication rates per cell for indicated modality.

(**D**), Violin plot of fraction of reads in peaks (FRiP) per cell for ChIP-seq, CUT&Tag and scMTR-seq for each histone modification. Numbers above each dataset shows mean values. Boxplots show median with interquartile range and minimum/maximum whiskers. Dot plots show the FRiP per cell for indicated modality.

(**E**), Table comparing scMTR-seq with other methods to profile histone modifications in single cells. Note that sequencing depth was not normalised across the different single-cell methods, and that the number of reads per cell is influenced by the sequencing depth.

(**F**), Number of peaks for each histone modification, defined using data from either ChIP-seq, CUT&Tag or scMTR-seq.

(**G**) and (**H**), Heatmaps show the percentage overlap between peaks defined using ENCODE ChIP-seq or single-target CUT&Tag and scMTR-seq for five histone modifications. The overlap is divided by the number of peaks of the data in each row.

(**I**), Genome browser tracks show histone modification signals for ENCODE ChIP-seq, single-target CUT&Tag, bulk sample multi-target CUT&Tag (six targets; “Bulk-MT”) and computationally aggregated scMTR-seq (six targets; “Aggregated MT”).

(**J**) and (**K**), Pearson correlation coefficients of histone modification signals of 5kb bin (blue) or 20kb (red) bin between aggregated data of different cell numbers of down-sampled scMTR-seq with corresponding histone modification data from single-modality CUT&Tag (**J**) or total scMTR-seq aggregated data (**K**).

Figure S5

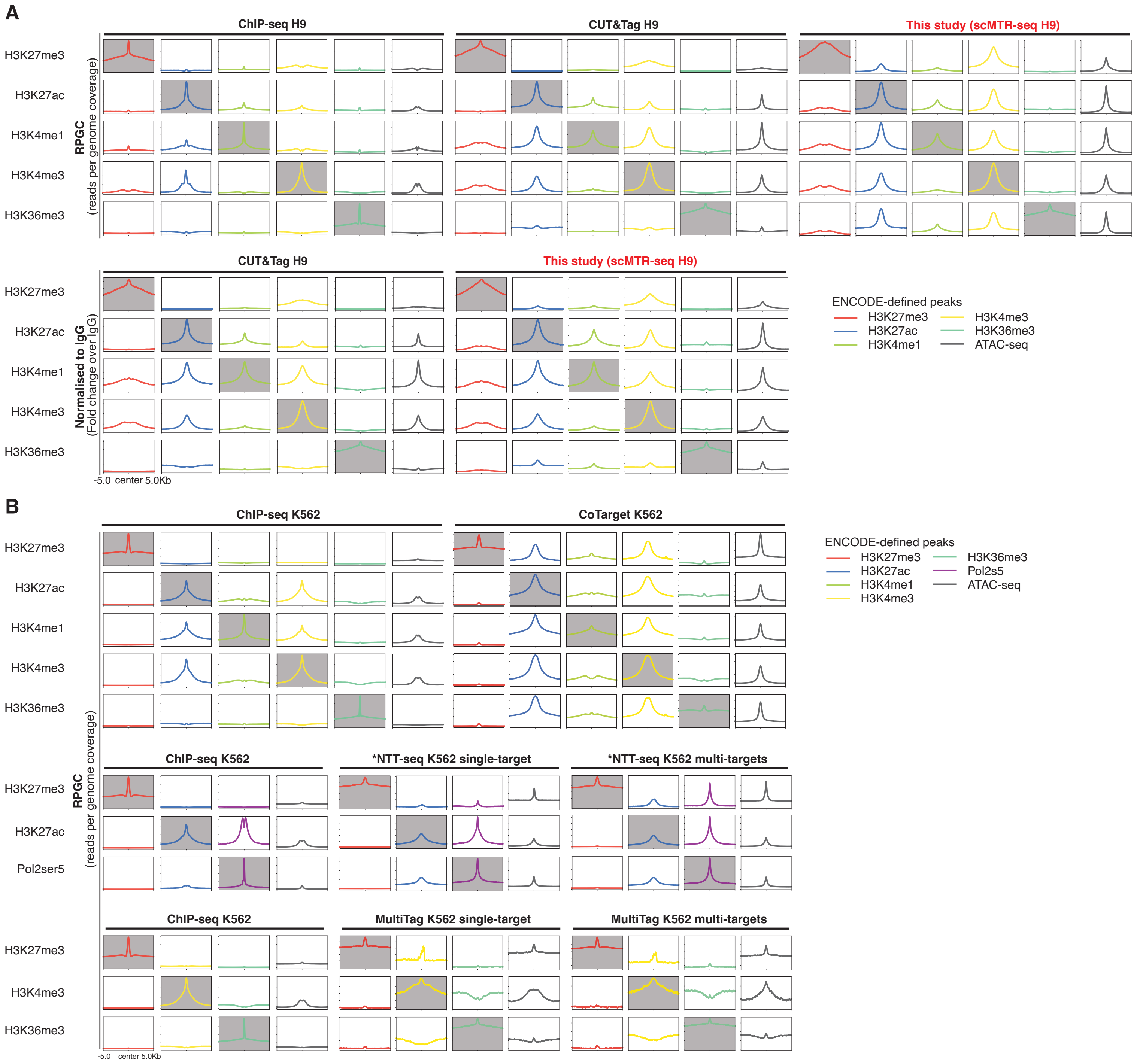

Notes:
1. Bigwig files are downloaded from GEO or ENCODE.
2. * files used hg38, the rest used hg19.

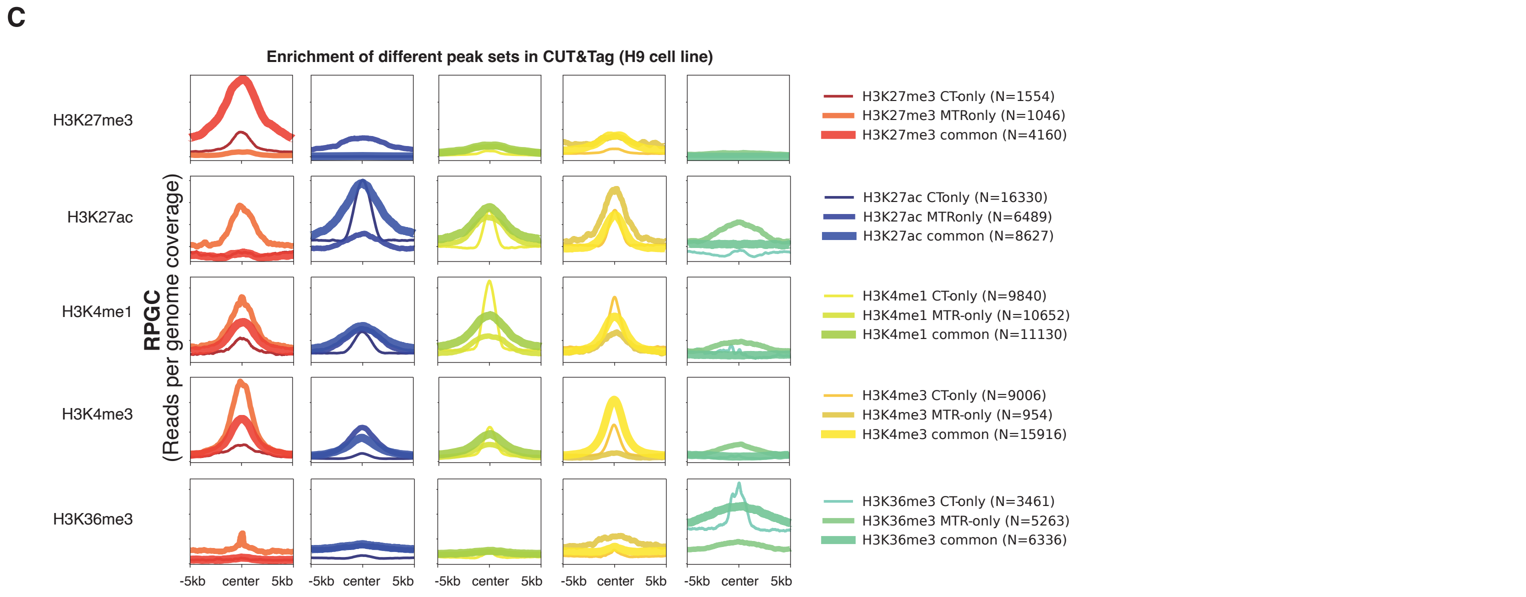

Fig. S5. Benchmarking of scMTR-seq.

(**A**), Line plots show enrichment of histone modification signals and IgG at ENCODE-defined ChIP-seq and ATAC-seq peaks. Signals are from ChIP-seq, single-target CUT&Tag and scMTR-seq experiments carried out on human pluripotent stem cells (H9 line). Plots underneath show the same CUT&Tag and scMTR-seq data normalised with integrated IgG signals.

(**B**), Similar plots to A, except that the data shown are obtained from other single-cell histone modification profiling methods (CoTarget, NTT-seq and MulTI-Tag) as well as ChIP-seq. These data are from K562 cells.

(**C**), Line plots show enrichment of histone modification signals in CUT&Tag assay at different peaks that are defined in CUT&Tag assay *only* ("CTonly"), scMTR-seq assay *only* ("MTRonly") or defined in *both* CUT&Tag and scMTR-seq assays ("common") for the same histone modification. N, peak numbers.

Figure S6

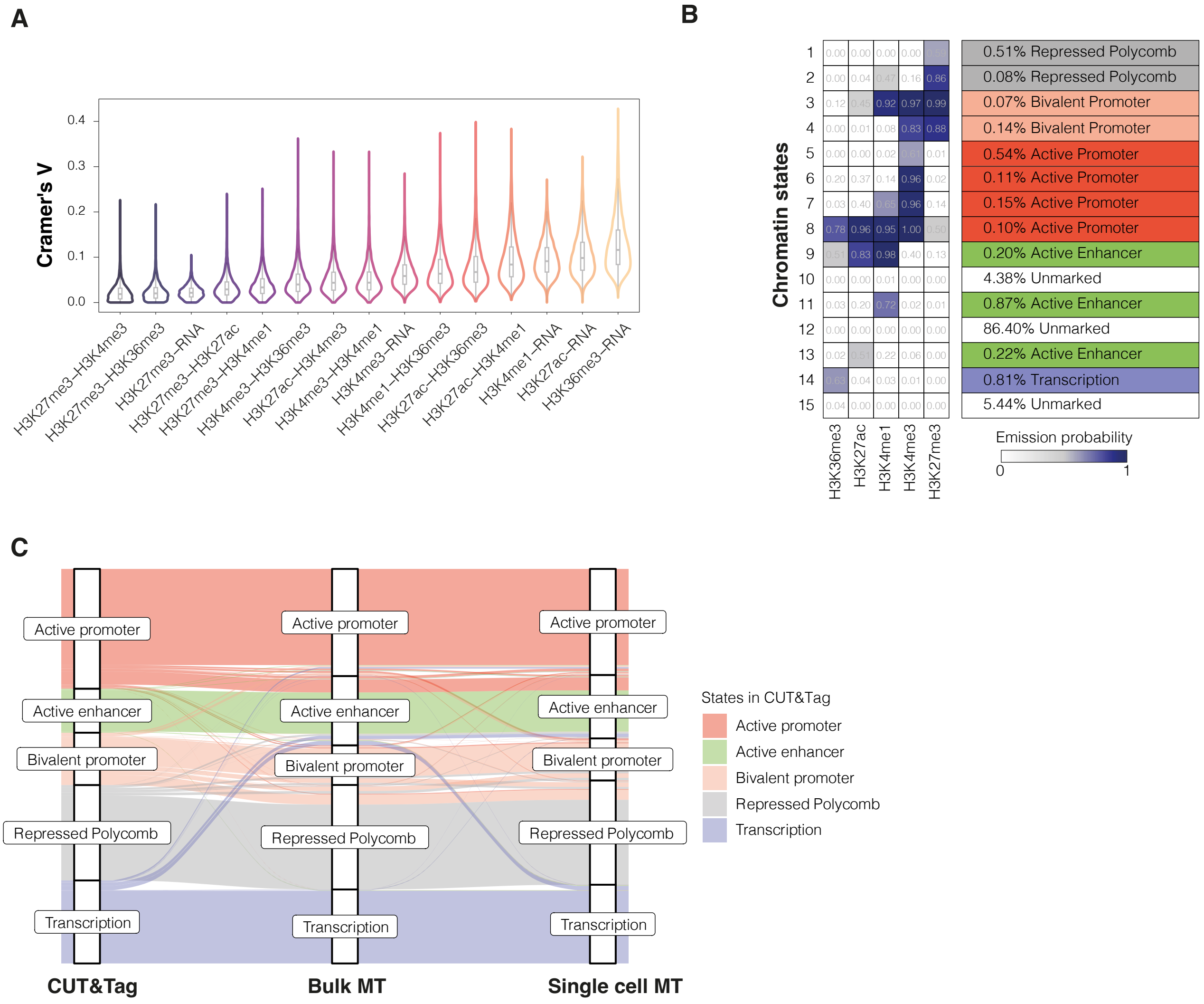
A
B
C
Cramer's V
0.0
0.1
0.2
0.3
0.4
H3K27me3–H3K4me3
H3K27me3–H3K36me3
H3K27me3–RNA
H3K27me3–H3K27ac
H3K27me3–H3K4me1
H3K4me3–H3K36me3
H3K27ac–H3K4me3
H3K4me3–H3K4me1
H3K4me3–RNA
H3K4me1–H3K36me3
H3K27ac–H3K36me3
H3K27ac–H3K4me1
H3K4me1–RNA
H3K27ac–RNA
H3K36me3–RNA
Chromatin states
0.51% Repressed Polycomb
0.08% Repressed Polycomb
0.07% Bivalent Promoter
0.14% Bivalent Promoter
0.54% Active Promoter
0.11% Active Promoter
0.15% Active Promoter
0.10% Active Promoter
0.20% Active Enhancer
4.38% Unmarked
0.87% Active Enhancer
86.40% Unmarked
0.22% Active Enhancer
0.81% Transcription
5.44% Unmarked
H3K36me3
H3K27ac
H3K4me1
H3K4me3
H3K27me3
Emission probability
0
1
Active promoter
Active enhancer
Bivalent promoter
Repressed Polycomb
Transcription
CUT&Tag
Bulk MT
Single cell MT
States in CUT&Tag
Active promoter
Active enhancer
Bivalent promoter
Repressed Polycomb
Transcription

Fig. S6. Analysis of histone modifications and chromatin states using scMTR-seq.

(**A**), Violin plots of Cramer's V show the relationship between each pair of histone modifications and with transcription. As expected, gene expression was positively associated with active histone marks, and H3K27me3 had low association with active histone marks and with gene expression. Boxplots are median with interquartile range and minimum/maximum whiskers.

(**B**), ChromHMM-defined chromatin states using computationally aggregated data from scMTR-seq. Numbers within squares refer to emission probability. Numbers within wider boxes refers to ratio of total genome.

(**C**), Plot shows the high overlap in regions defined in specific chromatin states as obtained from data generated by single-target CUT&Tag, bulk multi-target and scMTR-seq. MT, multi-target.

Figure S7

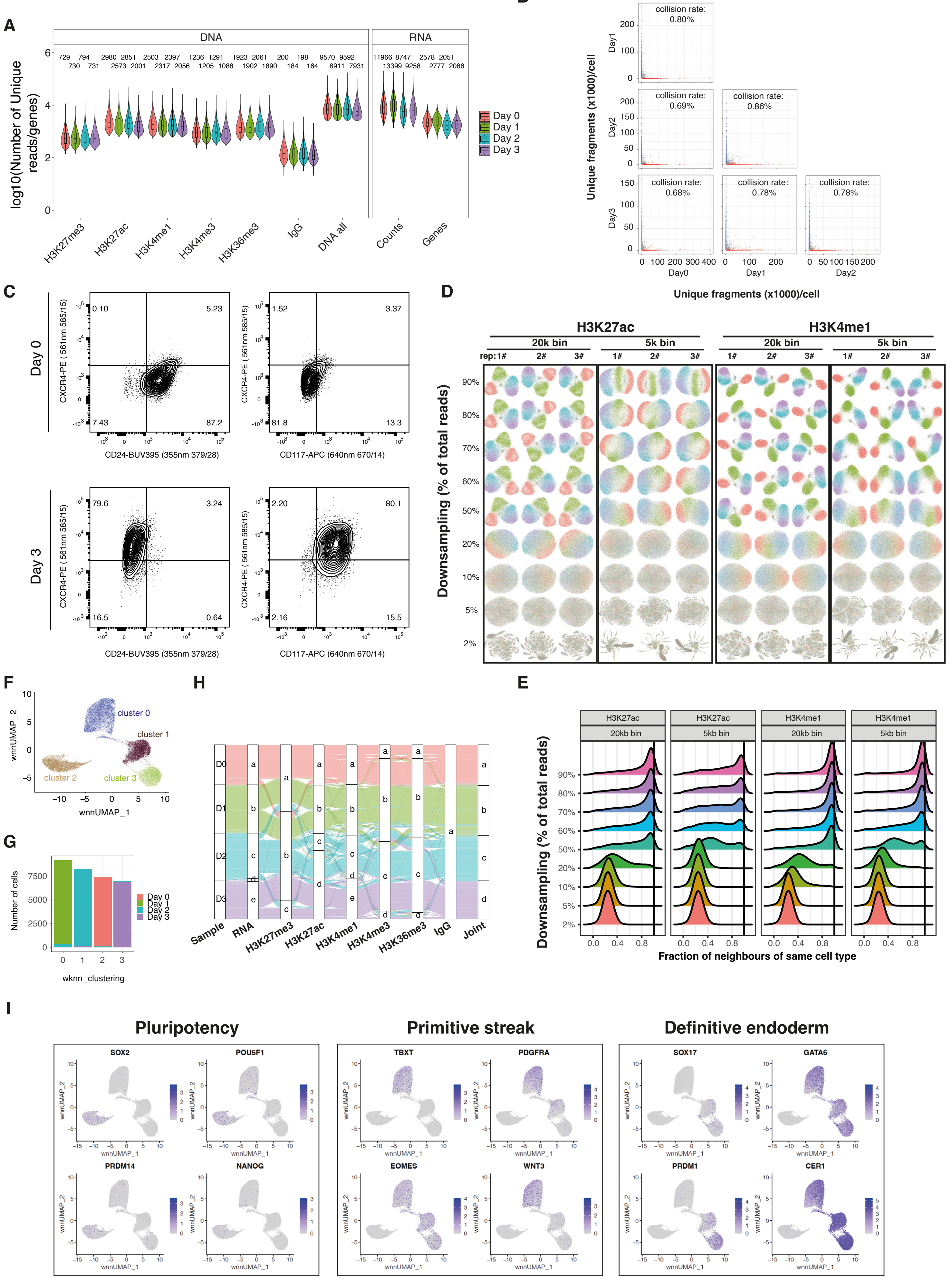

A
DNA
RNA
log10(Number of Unique reads/genes)
H3K27me3
H3K27ac
H3K4me1
H3K4me3
H3K36me3
IgG
DNA all
Counts
Genes
Day 0
Day 1
Day 2
Day 3
B
Unique fragments (x1000)/cell
Day0
Day1
Day2
Day3
collision rate: 0.80%
collision rate: 0.69%
collision rate: 0.86%
collision rate: 0.68%
collision rate: 0.78%
collision rate: 0.78%
C
Day 0
Day 3
CXCR4-PE (561nm 585/15)
CD24-BUV395 (355nm 379/28)
CD117-APC (640nm 670/14)
D
H3K27ac
H3K4me1
20k bin
5k bin
Downsampling (% of total reads)
E
Fraction of neighbours of same cell type
F
wnnUMAP_1
wnnUMAP_2
cluster 0
cluster 1
cluster 2
cluster 3
G
Number of cells
wknn_clustering
H
Sample
RNA
Joint
I
Pluripotency
Primitive streak
Definitive endoderm
SOX2
POU5F1
PRDM14
NANOG
TBXT
PDGFRA
EOMES
WNT3
SOX17
GATA6
PRDM1
CER1

Fig. S7. Multi-omic histone modification profiling of definitive endoderm differentiation.

(**A**), Violin plot of unique reads per cell for the six targets profiled and of unique RNA counts plus the number of genes per cell for the transcriptome. Numbers above each dataset shows mean values. Boxplots show median with interquartile range and minimum/maximum whiskers.

(**B**), Scatter plots show the very low collision rates (the rate that multiple cells may be indexed with the same barcode neglecting the unique sample ids introduced in RT step) using the single-cell multi-target method. This is visualised by comparing the number of unique fragments per cell id from different samples (days).

(**C**), Flow cytometry analysis of samples at day 0 and day 3 of endoderm differentiation.

(**D**), UMAPs of individual histone modification profiles with different downsampling ratios at genome-wide 20-kb bins and 5-kb bins. Three independent series of random downsampling analyses were performed.

(**E**), Fraction of cell nearest neighbours belonging to the same predicted cell type (based on RNA clustering results). Neighbour graphs were defined using downsampled histone modification at genome-wide 20-kb bins and 5-kb bins. Three independent series of random downsampling results were merged.

(**F**), UMAP of all joint modalities to identify cell clusters.

(**G**), Distribution of samples at each timepoint for each cell cluster with all joint modalities.

(**H**), Distribution of cell timepoints and clusters by different modality as indicated.

(**I**), UMAPs with heatmap overlay show transcript expression for indicated genes.

Figure S8

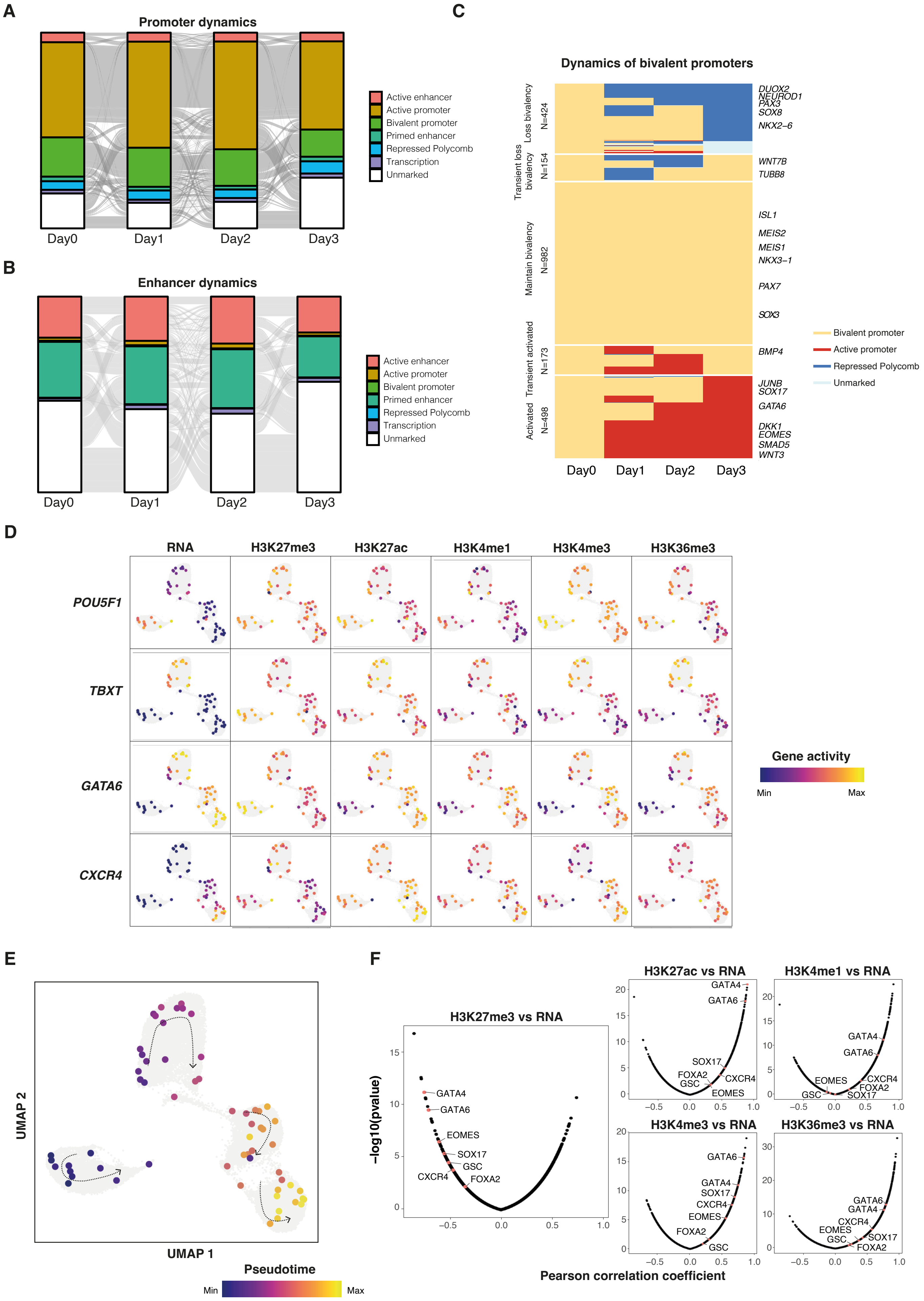
A
Promoter dynamics
Day0
Day1
Day2
Day3
Active enhancer
Active promoter
Bivalent promoter
Primed enhancer
Repressed Polycomb
Transcription
Unmarked
B
Enhancer dynamics
C
Dynamics of bivalent promoters
Loss bivalency N=424
Transient loss bivalency N=154
Maintain bivalency N=982
Transient activated N=173
Activated N=498
DUOX2
NEUROD1
PAX3
SOX8
NKX2-6
WNT7B
TUBB8
ISL1
MEIS2
MEIS1
NKX3-1
PAX7
SOX3
BMP4
JUNB
SOX17
GATA6
DKK1
EOMES
SMAD5
WNT3
Bivalent promoter
Active promoter
Repressed Polycomb
Unmarked
D
RNA
H3K27me3
H3K27ac
H3K4me1
H3K4me3
H3K36me3
POU5F1
TBXT
GATA6
CXCR4
Gene activity
Min
Max
E
UMAP 2
UMAP 1
Pseudotime
F
H3K27me3 vs RNA
H3K27ac vs RNA
H3K4me1 vs RNA
H3K4me3 vs RNA
H3K36me3 vs RNA
−log10(pvalue)
Pearson correlation coefficient
GATA4
GATA6
EOMES
SOX17
GSC
CXCR4
FOXA2

Fig. S8. Chromatin state dynamics in human definitive endoderm differentiation.

(**A**) and (**B**), Chromatin state annotations and alluvial plots of state transitions for (**A**) promoters and (**B**) enhancers over the endoderm differentiation timecourse.

(**C**), Heatmap shows behaviours of bivalent gene promoters over endoderm differentiation.

(**D**), UMAPs showing metacells, with heatmap overlay indicating the transcriptional activity of the indicated genes, along with the gene activities of each metacell for different histone modifications

(**E**), UMAP showing metacells with pseudotime path.

(**F**), Pearson correlation coefficient between gene activity of each histone modification with expression of gene in metacells. Each dot represents a gene.

Figure S9

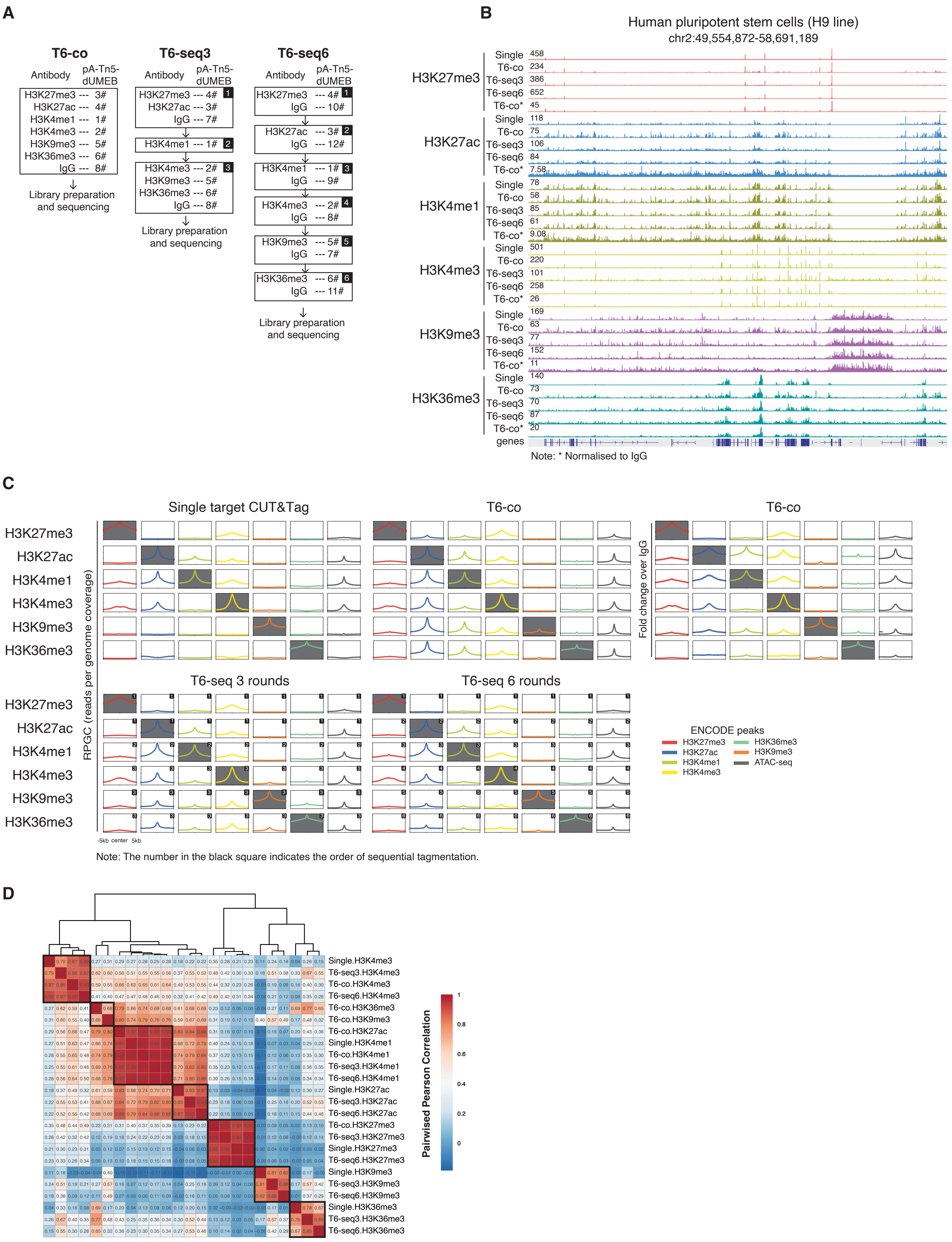

A
T6-co
Antibody
pA-Tn5-dUMEB
H3K27me3 --- 3#
H3K27ac --- 4#
H3K4me1 --- 1#
H3K4me3 --- 2#
H3K9me3 --- 5#
H3K36me3 --- 6#
IgG --- 8#
Library preparation and sequencing
T6-seq3
H3K27me3 --- 4#
H3K27ac --- 3#
IgG --- 7#
H3K4me1 --- 1#
H3K4me3 --- 2#
H3K9me3 --- 5#
H3K36me3 --- 6#
IgG --- 8#
T6-seq6
H3K27me3 --- 4#
IgG --- 10#
H3K27ac --- 3#
IgG --- 12#
H3K4me1 --- 1#
IgG --- 9#
H3K4me3 --- 2#
IgG --- 8#
H3K9me3 --- 5#
IgG --- 7#
H3K36me3 --- 6#
IgG --- 11#
B
Human pluripotent stem cells (H9 line)
chr2:49,554,872-58,691,189
H3K27me3
H3K27ac
H3K4me1
H3K4me3
H3K9me3
H3K36me3
Single
T6-co
T6-seq3
T6-seq6
T6-co*
genes
Note: * Normalised to IgG
C
Single target CUT&Tag
T6-co
T6-seq 3 rounds
T6-seq 6 rounds
RPGC (reads per genome coverage)
Fold change over IgG
-5kb center 5kb
ENCODE peaks
H3K27me3
H3K27ac
H3K4me1
H3K4me3
H3K36me3
H3K9me3
ATAC-seq
Note: The number in the black square indicates the order of sequential tagmentation.
D
Single.H3K4me3
T6-seq3.H3K4me3
T6-co.H3K4me3
T6-seq6.H3K4me3
T6-co.H3K36me3
T6-co.H3K9me3
T6-co.H3K27ac
Single.H3K4me1
T6-co.H3K4me1
T6-seq3.H3K4me1
T6-seq6.H3K4me1
Single.H3K27ac
T6-seq3.H3K27ac
T6-seq6.H3K27ac
T6-co.H3K27me3
T6-seq3.H3K27me3
Single.H3K27me3
T6-seq6.H3K27me3
Single.H3K9me3
T6-seq3.H3K9me3
T6-seq6.H3K9me3
Single.H3K36me3
T6-seq3.H3K36me3
T6-seq6.H3K36me3
Pairwised Pearson Correlation

Fig. S9. Sequential scMTR-seq.

(**A**), Experimental overview showing different strategies developed in this study to profile six histone modification from the same sample. Different antibodies were incubated with a uniquely-indexed pA-Tn5-dUMEB and blocked with normal IgG antibodies. Blocked antibody-pA-Tn5-dUMEB complexes were pooled as showed in the box for simultaneous tagmentation. T6-co, simultaneously targeting six histone modifications and IgG control; T6-seq3, targeting six histone modifications and IgG in three sequential rounds of tagmentation; T6-seq6, targeting six histone modifications and IgG in six sequential rounds tagmentation. Number in black square indicates the order of tagmentation.

(**B**), Genome browser tracks of histone modification signals in human pluripotent stem cells. Tracks correspond to single-target, multi-target T6-co, T6-seq3 and T6-seq6. The T6-co track is also shown normalised to IgG signal (T6-co*).

(**C**), Line plots show enrichment of histone modification signals at ENCODE-defined ChIP-seq and ATAC-seq peaks. Signals are from single-target CUT&Tag, as well as variations of scMTR-seq including multi-target T6-co, T6-seq3 and T6-seq6. The T6-co panel on the right is normalised to IgG signal. The number in the black square shows the order in the sequential tagmentation.

(**D**), Heatmaps show pairwise Pearson correlation between datasets from single-target CUT&Tag, multi-targets T6-co, T6-seq3 and T6-seq6 assays using genome-wide signals of 5-kb bins. The numbers show the Pearson correlation coefficients.

Figure S10

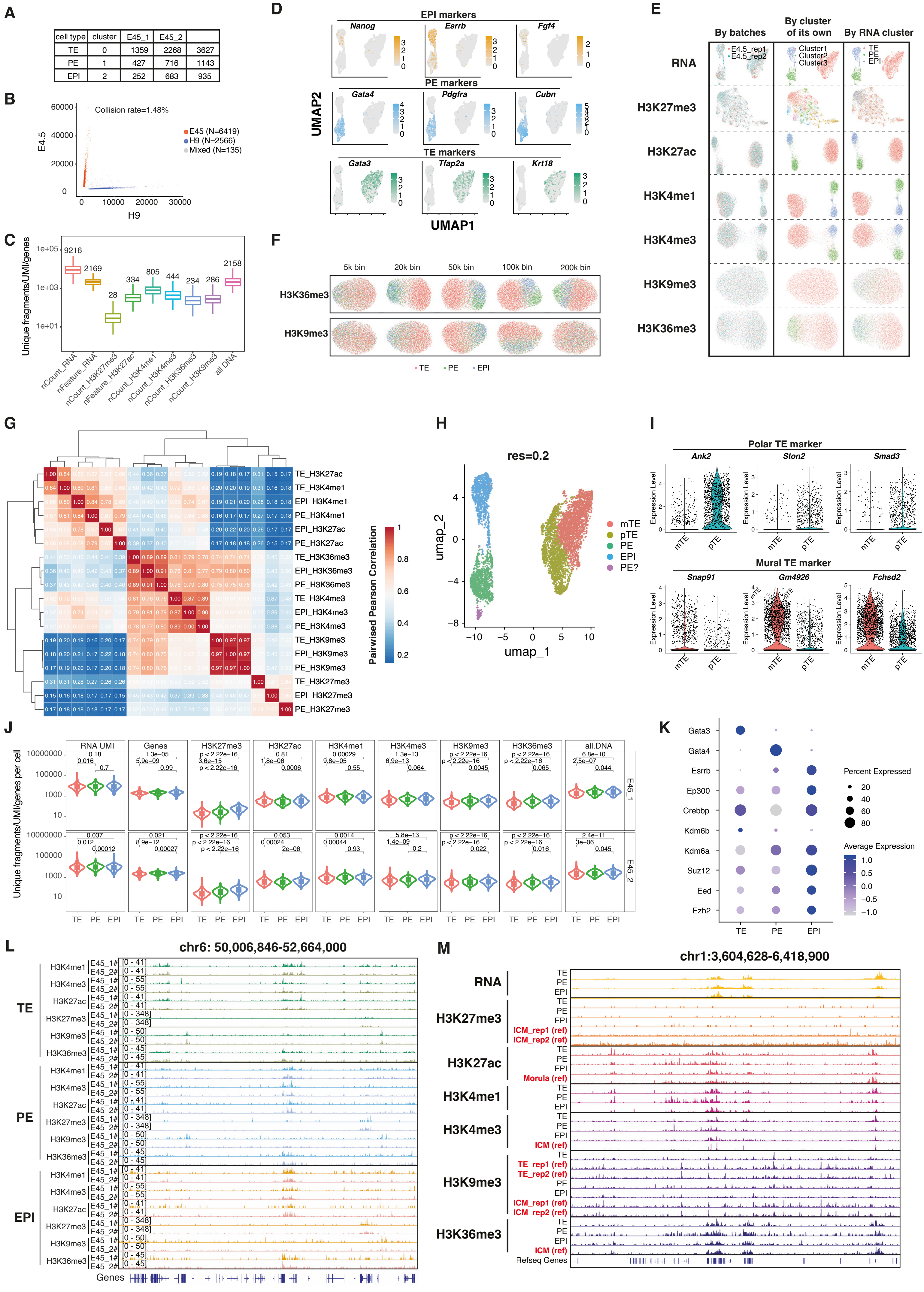
A
cell type | cluster | E45_1 | E45_2
TE | 0 | 1359 | 2268 | 3627
PE | 1 | 427 | 716 | 1143
EPI | 2 | 252 | 683 | 935
B
Collision rate=1.48%
E45 (N=6419)
H9 (N=2566)
Mixed (N=135)
E4.5
H9
C
Unique fragments/UMI/genes
9216
2169
28
334
805
444
234
286
2158
nCount_RNA
nFeature_RNA
nCount_H3K27me3
nFeature_H3K27ac
nCount_H3K4me1
nCount_H3K4me3
nCount_H3K36me3
nCount_H3K9me3
all.DNA
D
EPI markers
Nanog
Esrrb
Fgf4
PE markers
Gata4
Pdgfra
Cubn
TE markers
Gata3
Tfap2a
Krt18
UMAP2
UMAP1
E
By batches
By cluster of its own
By RNA cluster
RNA
H3K27me3
H3K27ac
H3K4me1
H3K4me3
H3K9me3
H3K36me3
E4.5_rep1
E4.5_rep2
Cluster1
Cluster2
Cluster3
TE
PE
EPI
F
5k bin
20k bin
50k bin
100k bin
200k bin
H3K36me3
H3K9me3
TE
PE
EPI
G
TE_H3K27ac
TE_H3K4me1
EPI_H3K4me1
PE_H3K4me1
EPI_H3K27ac
PE_H3K27ac
TE_H3K36me3
EPI_H3K36me3
PE_H3K36me3
TE_H3K4me3
EPI_H3K4me3
PE_H3K4me3
TE_H3K9me3
EPI_H3K9me3
PE_H3K9me3
TE_H3K27me3
EPI_H3K27me3
PE_H3K27me3
Pairwised Pearson Correlation
H
res=0.2
umap_2
umap_1
mTE
pTE
PE
EPI
PE?
I
Polar TE marker
Ank2
Ston2
Smad3
Mural TE marker
Snap91
Gm4926
Fchsd2
Expression Level
mTE
pTE
J
RNA UMI
Genes
H3K27me3
H3K27ac
H3K4me1
H3K4me3
H3K9me3
H3K36me3
all.DNA
Unique fragments/UMI/genes per cell
E45_1
E45_2
TE
PE
EPI
K
Gata3
Gata4
Esrrb
Ep300
Crebbp
Kdm6b
Kdm6a
Suz12
Eed
Ezh2
Percent Expressed
Average Expression
TE
PE
EPI
L
chr6: 50,006,846-52,664,000
TE
PE
EPI
H3K4me1
H3K4me3
H3K27ac
H3K27me3
H3K9me3
H3K36me3
E45_1#
E45_2#
Genes
M
chr1:3,604,628-6,418,900
RNA
H3K27me3
H3K27ac
H3K4me1
H3K4me3
H3K9me3
H3K36me3
ICM_rep1 (ref)
ICM_rep2 (ref)
Morula (ref)
ICM (ref)
TE_rep1 (ref)
TE_rep2 (ref)
Refseq Genes

Fig. S10. scMTR-seq profiles six histone modifications and transcriptome of E4.5 embryos.
(**A**), Number of cells for each lineage and cluster defined by RNA data in two batches of scMTR-seq datasets.
(**B**), Collision rate: scatter plot shows the reads mapped to human genome and mouse genome. Mixed cells are defined by the percentage of specific genome mapped reads less than 80% of total reads.
(**C**), Unique fragments, UMI and genes per cell. Number labelled on top of the boxplot shows the average value.
(**D**), UMAPs show the expression level of different markers.
(**E**), UMAPs of each histone modification and RNA data using E4.5 blastocyst single cell data. Cells from different batches, clusters and lineages are shown in different colour as indicated.
(**F**), UMAPs of histone modifications data with genome-wide signals of different bin sizes. Lineages are shown in different colours.
(**G**), Pearson correlation using genome-wide 20kb bin data between different histone modifications and lineages.
(**H**), UMAP of RNA data with res=0.2 for defining clusters. Clusters are shown in different colours. mTE, mural trophectoderm; pTE, polar trophectoderm.
(**I**), Violin plots show RNA expression levels of gene markers for polar TE and mural TE.
(**J**), Violin plots show the unique fragments, UMI and genes of different lineages. Boxplots are median with interquartile range and minimum/maximum whiskers. P values are shown, two-sided Wilcox-test.
(**K**), Dot plot shows the expression of different H3K27me3 related modifiers in different lineages.
(**L**), Genome browser tracks show histone modification signals from two biological replicates.
(**M**), Genome browser tracks show RNA and histone modification data together with indicated published datasets. Published datasets are: H3K27me3 (GSE76687), H3K27ac (GSE207222), H3K4me3 (GSE71434), H3K9me3 (GSE97778), H3K36me3 (GSE112835).

Figure S11

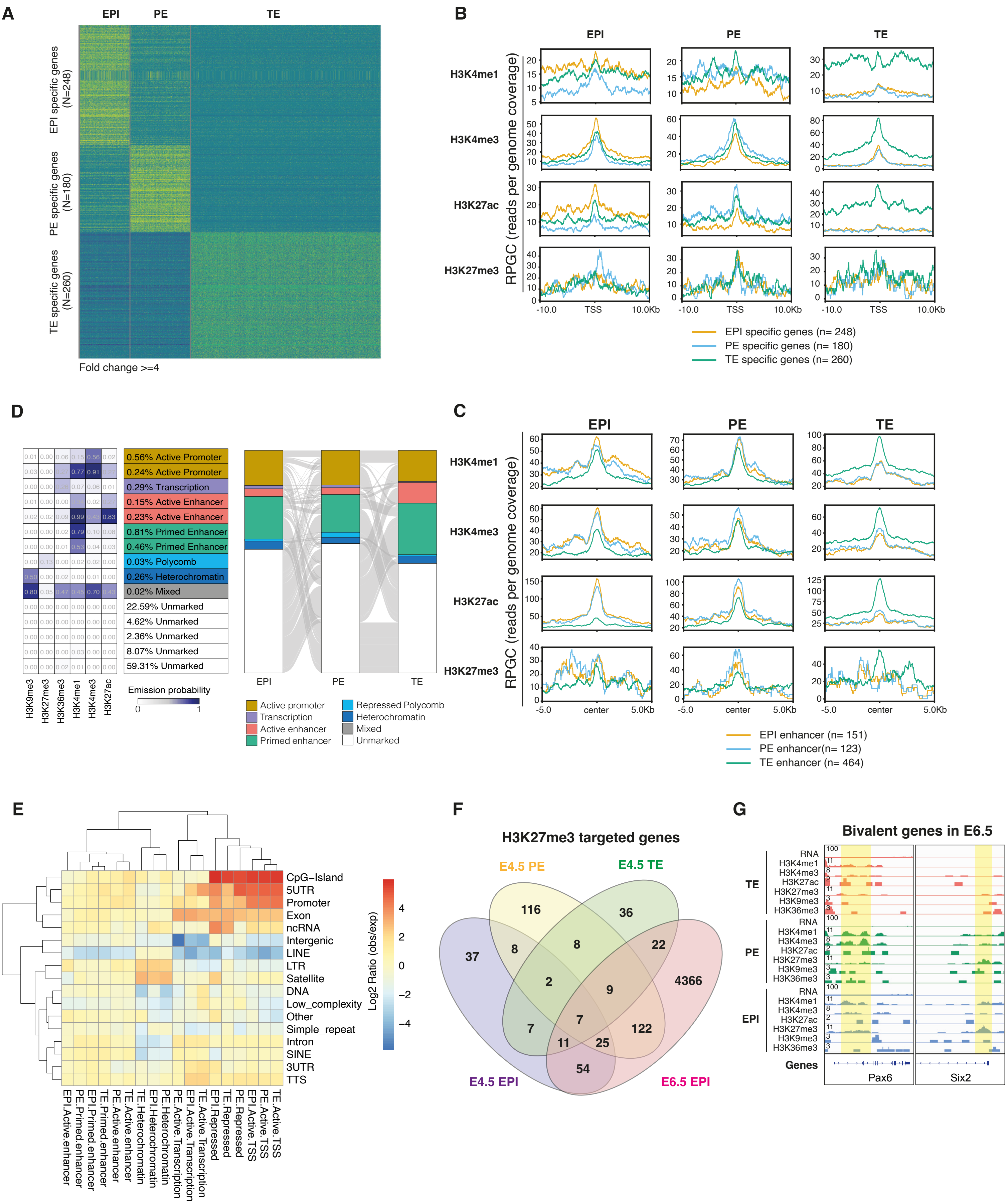
A
EPI
PE
TE
EPI specific genes (N=248)
PE specific genes (N=180)
TE specific genes (N=260)
Fold change >=4
B
H3K4me1
H3K4me3
H3K27ac
H3K27me3
RPGC (reads per genome coverage)
-10.0
TSS
10.0Kb
EPI specific genes (n= 248)
PE specific genes (n= 180)
TE specific genes (n= 260)
D
0.56% Active Promoter
0.24% Active Promoter
0.29% Transcription
0.15% Active Enhancer
0.23% Active Enhancer
0.81% Primed Enhancer
0.46% Primed Enhancer
0.03% Polycomb
0.26% Heterochromatin
0.02% Mixed
22.59% Unmarked
4.62% Unmarked
2.36% Unmarked
8.07% Unmarked
59.31% Unmarked
H3K9me3
H3K27me3
H3K36me3
H3K4me1
H3K4me3
H3K27ac
Emission probability
Active promoter
Transcription
Active enhancer
Primed enhancer
Repressed Polycomb
Heterochromatin
Mixed
Unmarked
C
-5.0
center
5.0Kb
EPI enhancer (n= 151)
PE enhancer(n= 123)
TE enhancer (n= 464)
E
CpG−Island
5UTR
Promoter
Exon
ncRNA
Intergenic
LINE
LTR
Satellite
DNA
Low_complexity
Other
Simple_repeat
Intron
SINE
3UTR
TTS
Log2 Ratio (obs/exp)
EPI.Active.enhancer
PE.Primed.enhancer
EPI.Primed.enhancer
TE.Primed.enhancer
PE.Active.enhancer
TE.Active.enhancer
TE.Heterochromatin
EPI.Heterochromatin
PE.Heterochromatin
PE.Active.Transcription
EPI.Active.Transcription
TE.Active.Transcription
EPI.Repressed
TE.Repressed
PE.Repressed
EPI.Active.TSS
PE.Active.TSS
TE.Active.TSS
F
H3K27me3 targeted genes
E4.5 PE
E4.5 TE
E4.5 EPI
E6.5 EPI
116
36
37
8
8
22
2
9
4366
7
7
122
11
25
54
G
Bivalent genes in E6.5
RNA
H3K4me1
H3K4me3
H3K27ac
H3K27me3
H3K9me3
H3K36me3
Genes
Pax6
Six2

Fig. S11. Lineage specific histone modifications in mouse E4.5 blastocysts.

(**A**), Lineage-specific gene expression (fold change>=4) in single cells. N, number of genes for each lineage set.

(**B**) and (**C**), Average signal of histone modifications (H3K4me1, H3K4me3, H3K27ac and H3K27me3) upstream (10kb) and downstream (-10kb) from TSS (**B**) upstream (5kb) and downstream (-5kb) from enhancer (**C**) in different lineage cells (EPI, epiblast; PE, primitive endoderm; TE, trophectoderm) for different lineage specific gene sets. N, number of TSS (**B**) or enhancer (**C**) for each lineage set.

(**D**), Left: heatmap shows emission probability of different histone modifications in different chromatin states; Middle: annotated chromatin states based on the combination of histone modifications and their genomic proportion; Right: alluvial plots show changes in chromatin states between different lineages. The numbers in white show the emission probabilities (left); colour shows different chromatin states (middle and right).

(**E**), Heatmap show the enrichment of chromatin states over genomic elements for each lineage. Elements were retained if they had >10 regions in at least one chromatin state.

(**F**), Overlap of nearest Polycomb-repressed genes (close to TSS ±2kb) among different lineages and time points.

(**G**), Genome browser tracks show histone modification and transcriptome profiles in *Pax6* and *Six2*, two bivalent genes in E6.5 Epiblast. Yellow shades highlight the H3K27me3 enriched at promoter regions in E4.5 Epiblast.

Figure S12

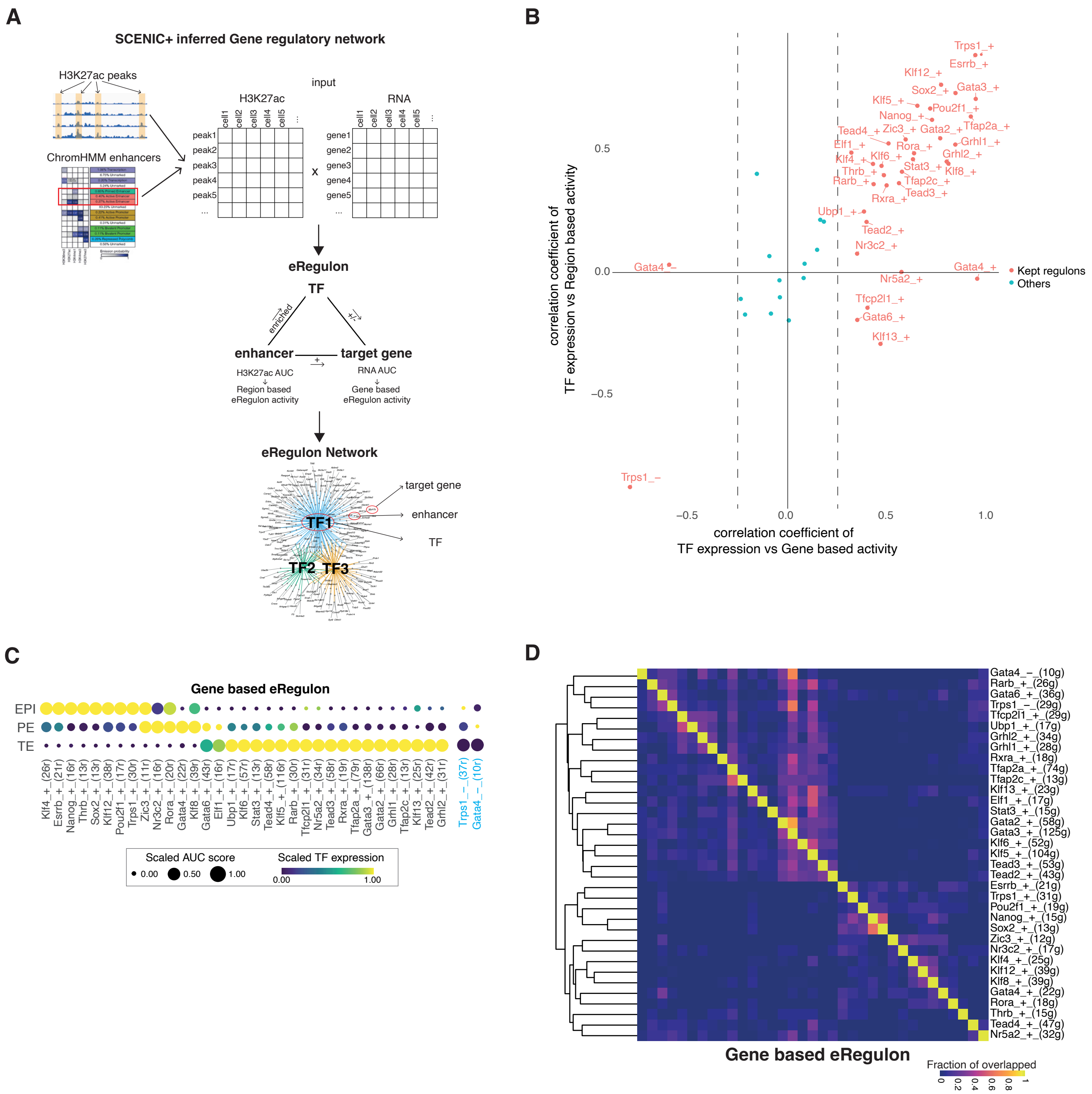

A
SCENIC+ inferred Gene regulatory network
H3K27ac peaks
ChromHMM enhancers
input
H3K27ac
RNA
cell1 cell2 cell3 cell4 cell5 ...
peak1 peak2 peak3 peak4 peak5 ...
gene1 gene2 gene3 gene4 gene5 ...
x
eRegulon
TF
enriched
enhancer
target gene
H3K27ac AUC
Region based eRegulon activity
RNA AUC
Gene based eRegulon activity
eRegulon Network
target gene
enhancer
TF
TF1
TF2
TF3
B
correlation coefficient of TF expression vs Region based activity
correlation coefficient of TF expression vs Gene based activity
Trps1_+
Esrrb_+
Klf12_+
Sox2_+
Gata3_+
Klf5_+
Pou2f1_+
Nanog_+
Tfap2a_+
Tead4_+
Zic3_+
Gata2_+
Grhl1_+
Elf1_+
Rora_+
Grhl2_+
Klf4_+
Klf6_+
Stat3_+
Klf8_+
Thrb_+
Rarb_+
Tfap2c_+
Tead3_+
Rxra_+
Ubp1_+
Tead2_+
Nr3c2_+
Gata4_–
Gata4_+
Nr5a2_+
Tfcp2l1_+
Gata6_+
Klf13_+
Trps1_–
Kept regulons
Others
C
Gene based eRegulon
EPI
PE
TE
Klf4_+_(26r)
Esrrb_+_(21r)
Nanog_+_(16r)
Thrb_+_(13r)
Sox2_+_(13r)
Klf12_+_(38r)
Pou2f1_+_(17r)
Trps1_+_(30r)
Zic3_+_(11r)
Nr3c2_+_(16r)
Rora_+_(20r)
Gata4_+_(22r)
Klf8_+_(39r)
Gata6_+_(43r)
Elf1_+_(16r)
Ubp1_+_(17r)
Klf6_+_(57r)
Stat3_+_(13r)
Tead4_+_(58r)
Klf5_+_(116r)
Rarb_+_(30r)
Tfcp2l1_+_(31r)
Nr5a2_+_(34r)
Tead3_+_(58r)
Rxra_+_(19r)
Tfap2a_+_(79r)
Gata3_+_(138r)
Gata2_+_(66r)
Grhl1_+_(28r)
Tfap2c_+_(13r)
Klf13_+_(25r)
Tead2_+_(42r)
Grhl2_+_(31r)
Trps1_–_(37r)
Gata4_–_(10r)
Scaled AUC score
0.00
0.50
1.00
Scaled TF expression
0.00
1.00
D
Gata4_–_(10g)
Rarb_+_(26g)
Gata6_+_(36g)
Trps1_–_(29g)
Tfcp2l1_+_(29g)
Ubp1_+_(17g)
Grhl2_+_(34g)
Grhl1_+_(28g)
Rxra_+_(18g)
Tfap2a_+_(74g)
Tfap2c_+_(13g)
Klf13_+_(23g)
Elf1_+_(17g)
Stat3_+_(15g)
Gata2_+_(58g)
Gata3_+_(125g)
Klf6_+_(52g)
Klf5_+_(104g)
Tead3_+_(53g)
Tead2_+_(43g)
Esrrb_+_(21g)
Trps1_+_(31g)
Pou2f1_+_(19g)
Nanog_+_(15g)
Sox2_+_(13g)
Zic3_+_(12g)
Nr3c2_+_(17g)
Klf4_+_(25g)
Klf12_+_(39g)
Klf8_+_(39g)
Gata4_+_(22g)
Rora_+_(18g)
Thrb_+_(15g)
Tead4_+_(47g)
Nr5a2_+_(32g)
Gene based eRegulon
Fraction of overlapped

Fig. S12. SCENIC+ infers gene regulatory network in mouse E4.5 blastocyst.

(**A**), Summary of approach using SCENIC+ with scMTR-seq data to infer enhancer-driven gene regulatory networks.

(**B**), Plot shows the correlation coefficient of transcription factor expression and activity of enhancer-target genes on x-axis, and the correlation coefficient of transcription factor expression and activity of target enhancers on y-axis.

(**C**), The TF expression and activity of eRegulons identified by SCENIC+ in different lineage of E4.5 blastocyst. The TF expression of the eRegulon on a colour scale, and activity of the eRegulon based on the enrichment of target genes on a size scale. +/- indicates whether transcription factor expression positively or negatively correlates with the expression of eRegulon target genes, respectively. The number of regions within each eRegulon are in brackets.

(**D**), Overlap of target genes of eRegulons. The overlap is divided by the number of target genes of the eRegulon in each row.

Table S1

scMTR-seq oligonucleotides and reagents. This table displays the sequences of oligonucleotides (**sheet 1**) and reagents (**sheet 2**) used in this study.

Table S2

Single cell metadata. This table displays scMTR-seq single cell data information.

Sheet 1. WA09/H9 endoderm differentiation scMTR-seq single cell data information; columns contain: single cell ids (“new.cellid”), sublibrary ids ("sub.lib”), time points (“timepoints”), cell types based on the timepoints (“celltype”), raw sequenced reads (“H3K27ac.allreads”, “H3K27me3.allreads”, “H3K36me3.allreads”, “H3K4me1.allreads”, “H3K4me3.allreads”, “RNA.allreads”, “rbIgG.allreads”), mapped reads (“H3K27ac.mappedreads”, “H3K27me3.mappedreads”, “H3K36me3.mappedreads”, “H3K4me1.mappedreads”, “H3K4me3.mappedreads”, “RNA.mappedreads”, “rbIgG.mappedreads”), unique reads (“nCount_RNA”, “nCount_H3K27me3”, “nCount_H3K27ac”, “nCount_H3K4me1”, “nCount_H3K4me3”, “nCount_H3K36me3”, “nCount_IgG”), unique genes (“nFeature_RNA”), percentage of mitochondrial RNA (“percent.mt”).

Sheet 2. E4.5 mouse blastocyst scMTR-seq single cell data information; columns contain: single cell ids (“new.cellid”), sublibrary ids ("sub.lib”), batchs (“batch”), cell types based on the RNA clustering (“celltype”), raw sequenced reads (“H3K27ac.allreads”,“H3K27me3.allreads”, “H3K36me3.allreads”, “H3K4me1.allreads”, “H3K4me3.allreads”, “H3K9me3.allreads”, “IgG1.allreads”, “IgG2.allreads”, “RNA.allreads”), mapped reads, (“H3K27ac.mappedreads”, “H3K27me3.mappedreads”, “H3K36me3.mappedreads”, “H3K4me1.mappedreads”, “H3K4me3.mappedreads”, , “H3K9me3.mappedreads”, “IgG1.mappedreads”, “IgG2.mappedreads”, “RNA.mappedreads”), unique reads (“nCount_RNA”,“nCount_H3K27me3”, “nCount_H3K27ac”, “nCount_H3K4me1”, “nCount_H3K4me3”, “nCount_H3K36me3”, “nCount_H3K9me3”), unique genes (“nFeature_RNA”), percentage of mitochondrial RNA (“percent.mt”).

Table S3

E45.RNA_markers. This table displays RNA markers in EPI lineage, PE lineage and TE lineage in E4.5 mouse blastocysts. Columns contain: gene name (“gene”), p value (“p_val”), log2 fold change of the average expression between the two groups (“ave_log2FC”), the percentage of cells detecting the gene in the first group (“pct.1 ”), the percentage of cells detecting the gene in the second group (“pct.2 ”), adjusted p-value by Bonferroni correction using all genes in the dataset (“p_val_adj”).

Table S4

E45 SCENIC+ selected eRegulons. This table displays eRegulons information. Column contain: region information of eRegulon ("Region_signature_name"), gene information of eRegulon ("Gene_signature_name"), TF of eRegulon ("TF"), whether is extended motif ("is_extended"), regions of eRegulons ("Region"), targeting genes ("Gene"), region to gene importance score calculated by GBM method ("R2G_importance"), region to gene correlation calculated by SR method ("R2G_rho"), TF to gene importance score calculated by GBM method ("TF2G_importance"), TF to gene correlation calculated by SR method ("TF2G_rho"),("TF2G_importance_x_abs_rho"), name of eRegulon ("Consensus_name")

Table S5

E45 SCENIC+ ERegulon single cell activity. This table displays transcription factor expression level, region based Regulon activity and gene based regulon activity. Columns contain: Name of transcription factor ("TF"), cell id ("cell"), gene information of eRegulon ("Gene_signature_name"), region information of eRegulon ("Region_signature_name"), AUC enrichment score of H3K27ac on enhancer regions ("region.AUC"), AUC enrichment score of expression of downstream gene ("gene.AUC"), TF expression level ("EXP"), cell type by RNA annotation ("celltype").
